# GSEA-assisted gene signatures valid for combinations of prognostic markers in PCNSL

**DOI:** 10.1038/s41598-020-65463-6

**Published:** 2020-05-21

**Authors:** Yasuo Takashima, Momoko Hamano, Junya Fukai, Yasuo Iwadate, Koji Kajiwara, Tsutomu Kobayashi, Hiroaki Hondoh, Ryuya Yamanaka

**Affiliations:** 1 Osaka Iseikai Clinic for Cancer Therapy, Iseikai Holonics Group, Osaka, Japan; 2 Laboratory of Molecular Target Therapy for Cancer, Graduate School of Medical Science, Kyoto Prefectural University of Medicine, Kyoto, Japan; 3 Department of Bioscience and Bioinformatics, Faculty of Computer Science and Systems Engineering, Kyushu Institute of Technology, Iizuka, Fukuoka Japan; 4 Department of Neurological Surgery, Wakayama Medical University School of Medicine, Wakayama, Japan; 5 Department of Neurosurgery, Graduate School of Medical Sciences, Chiba University, Chiba, Japan; 6 Department of Neurosurgery, Graduate School of Medical Sciences, Yamaguchi University, Ube, Yamaguchi Japan; 7 Department of Neurosurgery, Toyama Prefectural Central Hospital, Toyama, Japan

**Keywords:** CNS cancer, Prognostic markers, Lymphoproliferative disorders

## Abstract

Primary central nervous system lymphoma (PCNSL) is a brain malignant non-Hodgkin’s B-cell lymphoma. The standard treatments are high-dose methotrexate (MTX)-based chemotherapies and deferred whole brain radiotherapy. However, MTX resistance-dependent global expression and signaling pathway changes and their relationship with prognoses have not yet been elucidated. Here, we conducted a global expression analysis with next-generation sequencing and gene set enrichment analysis (GSEA) in MTX-resistant PCNSL cell lines (HKBML-MTX and TK-MTX) and PCNSL tissues. In rank scores, genes listed in HKBML-MTX and TK-MTX were enriched in PCNSL with poor prognoses. In fold changes, a part of differentially-expressed genes in PCNSL tissues were also detected in HKBML-MTX and TK-MTX cells; *FOXD2-AS1* and *MMP19* were commonly expressed in both HKBML-MTX and TK-MTX, *FABP5* and *CD70* were HKBML-MTX-specifically expressed, and *CLCN2*, *HOXB9*, *INE1*, and *LRP5L* were TK-MTX-specifically expressed, which may provide a combination of prognostic markers on MTX-sensitivities in PCNSL. Additionally, PCNSL subgroups, divided with hierarchical clustering and Kaplan-Meier methods, included twenty commonly expressed genes in both HKBML-MTX and TK-MTX, ten HKBML-MTX-specifically expressed genes, and two TK-MTX-specifically expressed genes. These results suggest that the GSEA-assisted gene signatures can provide a combination for prognostic markers in recurrent PCNSL with MTX resistances.

## Introduction

Primary central nervous system lymphoma (PCNSL) is a rare subtype of cerebral malignant non-Hodgkin’s B-cell lymphoma with a median overall survival (OS) of approximately four years, which accounts for 3% of all primary brain tumors and 1% of non-Hodgkin’s lymphomas (NHLs) in adults^1,2^. Methotrexate (MTX) is an antifolate that inhibits dihydrofolate (DHF) reductase (DHFR) activity in purine and thymidine syntheses and regulates the expression of glucocorticoid receptors α and β in human blood cells *in vitro*^3,4^. High-dose MTX (HD-MTX) is used as a first-line treatment in PCNSL^5^. Second-line treatments are also required for 10–35% of patients with refractory diseases and for another 35–60% or more who have relapse-acquired MTX resistances^6^. Thus, almost PCNSLs recur with MTX resistances despite standard treatments^7^.

MTX is an antifolate that inhibits DNA synthesis^4^. DHFR converts DHF to tetrahydrofolate (THF), a basic form of reduced folate coenzymes^8^. MTX resistances are acquired through alternative intrinsic mechanisms in malignant cells, which include decreased drug transport due to mutations and reduced transcription activity of folate carrier genes, dysregulated DHFR activity and affinity, and decreased polyglutamination of MTX due to decreased folylpolyglutamate synthetase (FPGS) activity and increased γ-glutamyl hydrolase (GGH) activity^9–11^. MTX exposure induces the activity of methylenetetrahydrofolate dehydrogenase 1 (MTHFD1), serine hydroxymethyltransferase 1 (SHMT1), 5,10-methylenetetrahydrofolate reductase (MTHFR), 5-methyltetrahydrofolate-homocysteine methyltransferase (MTR), and methionine synthase reductase (MTRR), involved in the folate metabolism and intracellular resupply of THF^12^. A recent study demonstrate cell-type-specific intrinsic alterations and potential chemotherapies in NHL cell lines^13^. The 50% inhibition concentration (IC_50_) for MTX in MTX-resistant PCNSL cell line HKBML (HKBML-MTX) is markedly higher than that in MTX-resistant PCNSL cell line TK (TK-MTX) and MTX-resistant systemic diffuse large B-cell lymphoma (DLBCL) cell line Raji (Raji-MTX)^13^. Conversely, sensitivity to a molecular targeted drug, bortezomib (26 S proteasome inhibitor) is observed in TK-MTX, but not in HKBML-MTX and Raji-MTX, with expression changes of MTX and folate metabolism including GGH, DHFR, FPGS, thymidylate synthase (TYMS), and MTHFD1^13^. Although MTX-resistance-agitated global expression and signaling pathway changes associated with patient prognoses have not yet been elucidated, recent studies are gradually revealing cell-type specificities between HKBML and TK^13–15^. PCNSL is heterogenous and distinct from non-CNS DLBCL in compositions of immune cells including T and dendritic cells. ^16^ While, PCNSL reflects closely features of activated B-cell-type DLBCL (ABC-DLBCL)^16^.

Here, we conducted a global expression analysis with next-generation sequencing (NGS) and gene set enrichment analysis (GSEA) in HKBML-MTX, TK-MTX, and PCNSL clinical samples. A part of differentially expressed genes (DEGs) in HKBML-MTX and TK-MTX overlapped in PCNSL samples with poor prognosis. Further, commonly upregulated genes were detected in HKBML-MTX and TK-MTX, whereas most of downregulated genes were unique in the two cell lines. Conversely, a part of DEGs in PCNSL specimens were also detected in HKBML-MTX and TK-MTX. Particularly, expression patterns of downregulated genes in HKBML-MTX and TK-MTX separated survival curves of PCNSL subgroups. These results suggest that the GSEA-assisted gene signatures on precise expression profiling would be useful for prognosis prediction and refractory recurrence acquired-MTX resistances in PCNSL.

## Results

### GSEA in MTX-resistant PCNSL cells and clinical samples

**Figure 1:**
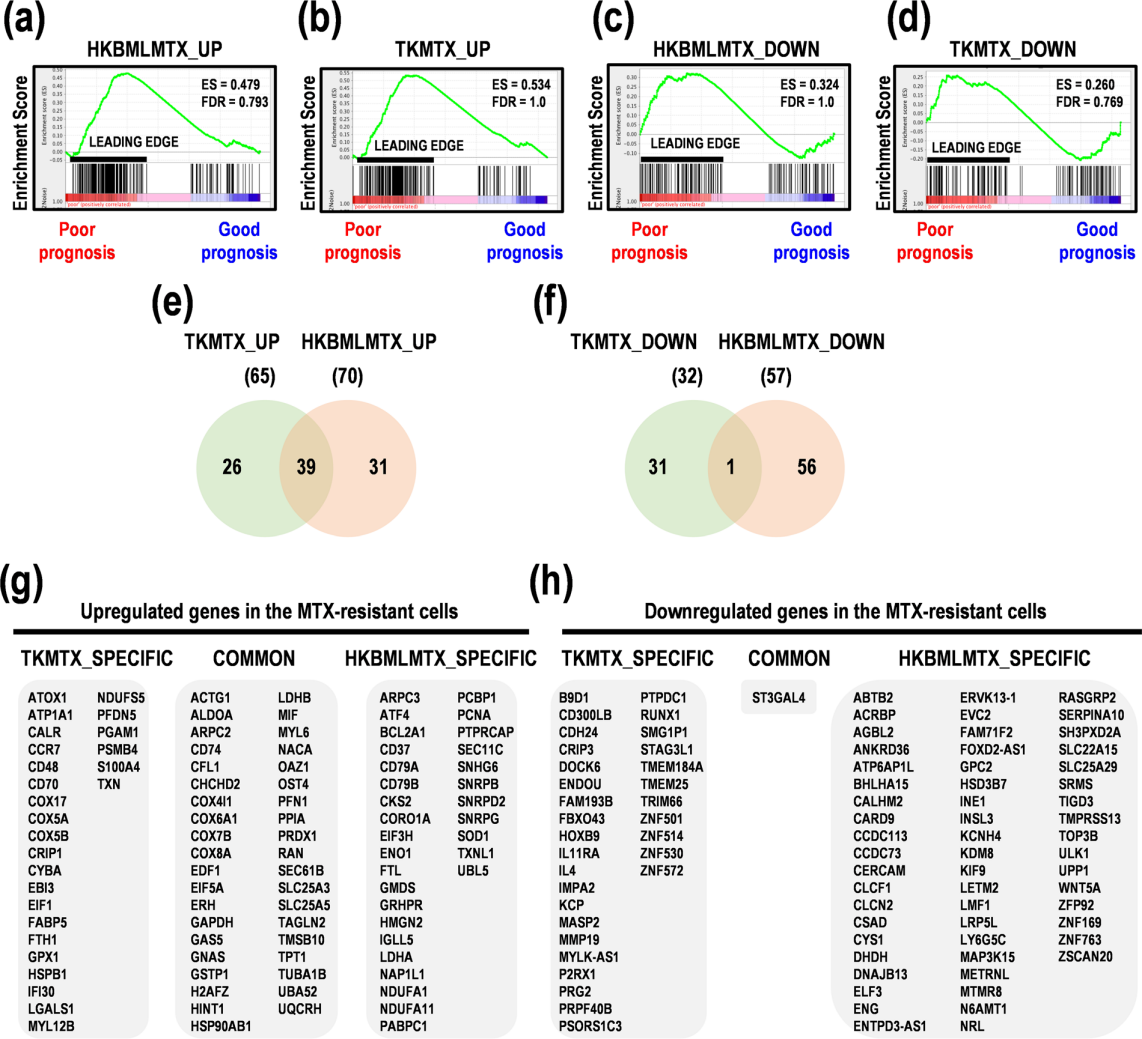
Gene set enrichment analysis (GSEA) in MTX-resistant PCNSL cells. (**a,b**) GSEA for upregulated genes of MTX-resistant PCNSL cells in PCNSL with poor and good prognoses. The upregulated genes in (**a**) HKBML-MTX and (**b**) TK-MTX, compared with HKBML and TK, respectively. (**c,d**) GSEA for downregulated genes of MTX-resistant PCNSL cells in poor and good prognoses. The downregulated genes in (**c**) HKBML-MTX and (**d**) TK-MTX, compared with HKBML and TK, respectively. ES; enrichment score. (**e,f**) Venn diagram of genes in MTX-resistant PCNSL cells, compared with control cells. Genes associated with high enrichment score in GSEA were selected. Numbers of genes were shown in Venn diagram. (**e**) The numbers of upregulated genes in HKBML-MTX and TK-MTX, compared with control cells. (**f**) The numbers of downregulated genes in HKBML-MTX and TK-MTX, compared with control cells. (**g,h**) Lists of cell-type-specifically and commonly expressed genes in HKBML-MTX and TK-MTX, compared with control cells, associated with ESs in GSEA. (**g**) Upregulated genes. (**h**) Downregulated genes.

In this study, to determine gene signatures in MTX-resistant PCNSL cells and PCNSL patients with poor prognoses, we conducted GSEA with NGS in PCNSL cell lines HKBML and TK and their MTX-resistant cells (HKBML-MTX and TK-MTX), and PCNSL tumor tissues. In rank scores, 200 upregulated genes in HKBML-MTX and TK-MTX, compared with HKBML and TK, respectively, were enriched into leading edges of PCNSL with poor prognoses. The top enrichment scores (ESs) were 0.479 and 0.534 in HKBML-MTX and TK-MTX, respectively (Fig. 1a,b, Suppl. Table S1). While, 200 downregulated genes in HKBML-MTX and TK-MTX, compared with HKBML and TK, respectively, were also enriched into leading edges of PCNSL with poor prognoses. The top ESs were 0.324 and 0.260 in HKBML-MTX and TK-MTX, respectively (Fig. 1c,d, Suppl. Table S1). These results indicate that the upregulated genes in HKBML-MTX and TK-MTX are strongly enriched in PCNSL with poor prognoses and also denote that a part of downregulated genes in HKBML-MTX and TK-MTX are inversely correlated with PCNSL with good prognoses, suggestive of malignancies in PCNSL.

### Rank score-associated genes of MTX-resistant PCNSL cells in GSEA

We next examined rank scored genes in GSEA, which was shown in Venn diagrams (Fig. 1e,f). The 39 genes were found in the upregulated gene sets in TK-MTX and HKBML-MTX (Fig. 1e,g, Suppl. Table S2). The upregulated genes in HKBML-MTX, compared with HKBML, included nicotinamide adenine dinucleotide (NAD)H dehydrogenase 1α subcomplex subunit 1 (*NDUFA1*) and 11 (*NDUFA11*) for mitochondrial electron transport, solute carrier family 25 member 5 (*SLC25A5)* for ADP/ATP exchange, protein tyrosine phosphatase receptor type C-associated protein (*PTPRCAP*) for protein phosphatase, cyclin-dependent kinases regulatory subunit 2 (*CKS2*) for cell cycle, and cluster of differentiation (CD) 79B (*CD79B*) as B lymphocyte antigen receptors, in addition to high mobility group nucleosomal binding domain 2 (*HMGN2*) and small nuclear ribonucleoprotein-associated proteins B and B’ (*SNRPB*). The upregulated genes in TK-MTX, compared with TK, included *CD70* as tumor necrosis factor (TNF) ligands, Epstein-Barr virus-induced gene 3 (*EBI3*) for activation of T, B, and myeloid cells, interferon γ-inducible protein 30 (*IFI30*) for major histocompatibility complex (MHC) class II-restricted antigen processing, cytochrome b-245 α chain (*CYBA*) for superoxide production and phagocytosis, and cytochrome c oxidase subunit 5B, mitochondrial (*COX5B*) as a subunit of complex IV in mitochondrial electron transport chain, and β-galactoside-binding lectin (*LGALS1*) for suppression of Th1 and Th17 helper T cells, in addition to fatty acid-binding protein 5 (*FABP5*) and ferritin heavy chain 1 (*FTH1*). The commonly upregulated genes in both HKBML-MTX and TK-MTX included thymosin β-10 (*TMSB10*) for organization of cytoskeleton, and macrophage migration inhibitory factor (*MIF*) for suppression of anti-inflammatory effects. Interestingly, of the downregulated genes, antisense RNAs of forkhead box transcription factor D2 (*FOXD2-AS1*) and myosin light chain kinase (*MYLK-AS1*) were included in both HKBML-MTX and TK-MTX, implying that the expression of *FOXD2* and *MYLK* allow. While, only the ST3 β-galactoside α-2,3-sialyltransferase 4 (*ST3GAL4*) gene as sialyltransferase for β-galactoside was common in the downregulated gene sets in both TK-MTX and HKBML-MTX (Fig. 1f,h, Suppl. Table S2). These results suggest that the upregulated or downregulated genes in HKBML-MTX and TK-MTX could be potential prognostic factors and estimation of molecular pathways for targeted therapy in PCNSL.

### GSEA-assisted genes as prognostic marker candidates in PCNSL

**Figure 2:**
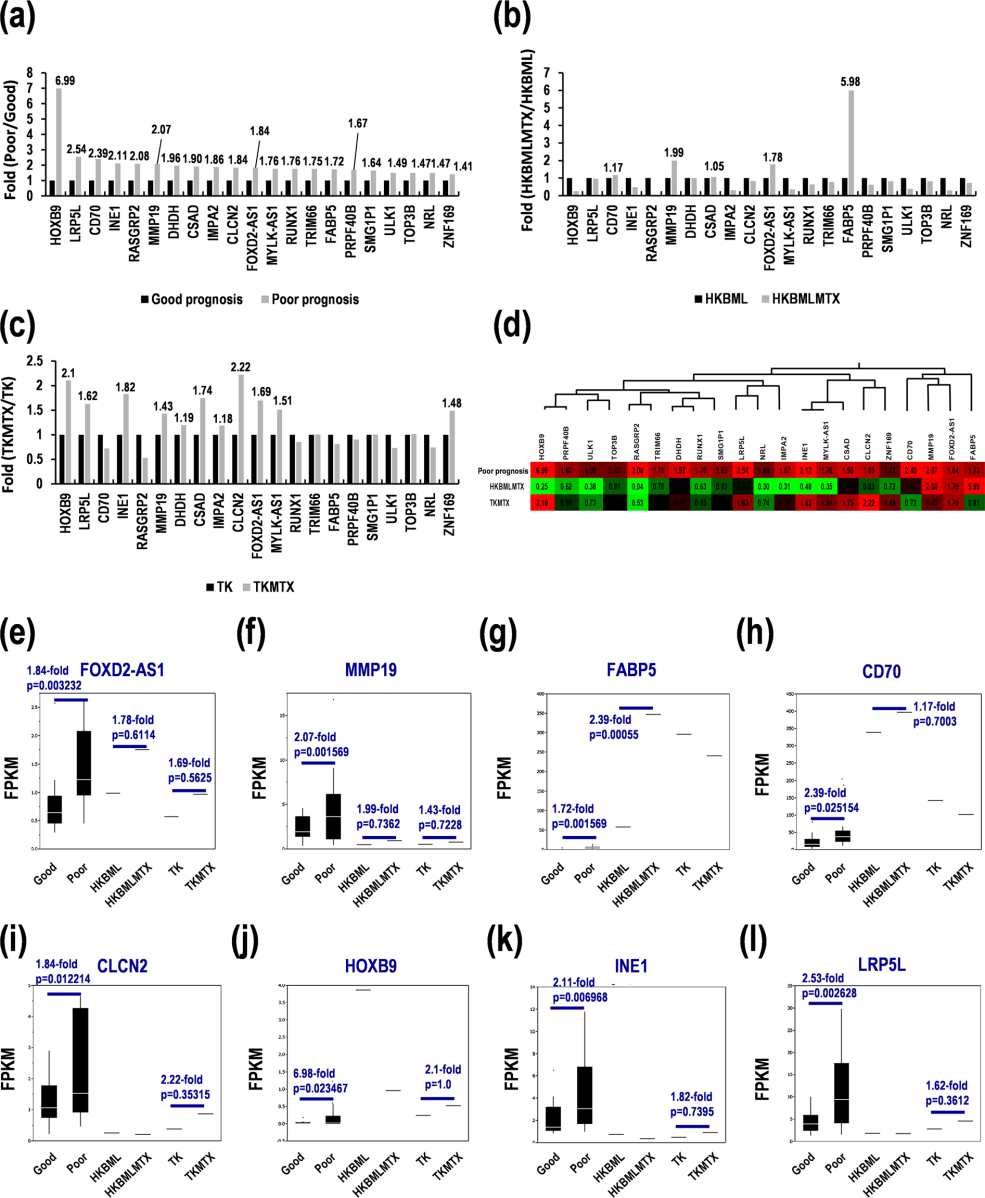
Identification of cell-type-dependent marker candidates for PCNSL with poor survivals and MTX resistances. (**a-c**) Differential expression in (**a**) PCNSL with poor survivals, (**b**) HKBML-MTX, and (**c**) TK-MTX. (**d**) Clustering of differential expression marker candidates. Green-black-red as low-median-high expression. (**e–l**) Expression in PCNSL samples divided by prognoses in box-whisker plots. (**e**) *FOXD2-AS1* and (**f**) *MMP19* as gene marker candidates for PCNSL with poor survivals. (**g**) *FABP5* and (**h**) *CD70* as gene marker candidates for HKBML-MTX. (**i**) *CLCN2*, (**j**) *HOXB9*, (**k**) *INE1*, and (**l**) *LRP5L* as gene marker candidates for TK- MTX. FPKM; fragments per kilobase of exon model per million reads mapped.

As described above, the rank score-associated genes successfully promoted gene marker candidates in PCNSL. Then, we further examined gene marker candidates along expression changes in the MTX-resistant PCNSL cells. In the GSEA-assisted genes, the 21 genes were differentially expressed with >1.5-fold (*p* < 0.05) in PCNSL with poor prognoses, compared with good prognoses (Fig. 2a, Table 1). Of these, *FABP5* (5.98-fold), matrix metalloproteinase-19 (*MMP19*) (1.99-fold), *FOXD2-AS1* (1.78-fold), and *CD70* (1.17-fold) were also upregulated in HKBML-MTX, compared with HKBML (Fig. 2b). Similarly, chloride channel protein 2 (*CLCN2*) (2.22-fold), homeobox B9 (*HOXB9*) (2.1-fold), putative inactivation escape 1 protein (*INE1*) (1.82-fold), cysteine sulfinic acid decarboxylase (*CSAD*) (1.74-fold), *FOXD2-AS1* (1.69-fold), low-density lipoprotein receptor-related protein 5-like protein (*LRP5L*) (1.62-fold), *MYLK-AS1* (1.51-fold), zinc finger protein 169 (*ZNF169*) (1.48-fold), *MMP19* (1.43-fold), dihydrodiol dehydrogenase (*DHDH*) (1.19-fold), and inositol monophosphatase 2 (*IMPA2*) (1.18-fold) were upregulated in TK-MTX, compared with TK (Fig. 2c). Hierarchical clustering analyses clarified three types of DEGs; commonly upregulated genes in both HKBML-MTX and TK-MTX, HKBML-MTX-specifically upregulated genes, and TK-MTX-specifically upregulated genes, associated with poor prognoses in PCNSL (Fig. 2d). *FOXD2-AS1* and *MMP19* were commonly upregulated in HKBML-MTX and TK-MTX (Fig. 2e,f). *FABP5* and *CD70* were HKBML-MTX-specifically upregulated (Fig. 2g,h). *CLCN2*, *HOXB9*, *INE1*, and *LRP5L* were TK-MTX-specifically upregulated (Fig. 1i–l). Similarly, *CSAD*, *MYLK-AS1*, *ZNF169*, *DHDH*, and *IMPA2* were upregulated in the MTX-resistant PCNSL cells (Suppl. Fig. S1). These genes might be used for prognosis prediction and recurrent CNS tumors with MTX resistances in PCNSL.

**Table 1:** Characterization of prognosis marker candidates in MTX-resistant PCNSL with poor prognoses.

Gene symbol	Gene name
**(Poor prognosis)**	
CSAD	cysteine Sulfinic Acid Decarboxylase
FOXD2-AS1	FOXD2 antisense RNA 1 (head to head)
MMP19	matrix metallopeptidase 19
**(HKBML-MTX type)**	
FABP5	fatty acid binding protein 5 (psoriasis-associated)
CD70	CD70 molecule
**(TK-MTX type)**	
CLCN2	chloride channel protein 2
DHDH	dihydrodiol dehydrogenase (dimeric)
HOXB9	homeobox B9
IMPA2	inositol(myo)-1(or 4)-monophosphatase 2
INE1	inactivation escape 1 (non-protein coding)
LRP5L	low density lipoprotein receptor-related protein 5-like
MYLK-AS1	MYLK antisense RNA 1
NRL	neural retina leucine zipper
PRPF40B	PRP40 pre-mRNA processing factor 40 homolog B (S. cerevisiae)
RASGRP2	RAS guanyl releasing protein 2 (calcium and DAG-regulated)
RUNX1	runt-related transcription factor 1
SMG1P1	SMG1 pseudogene 1
TOP3B	topoisomerase (DNA) III beta
TRIM66	tripartite motif containing 66
ULK1	unc-51 like autophagy activating kinase 1
ZNF169	zinc finger protein 169

### Differential expression of GSEA-assisted genes in PCNSL

**Figure 3:**
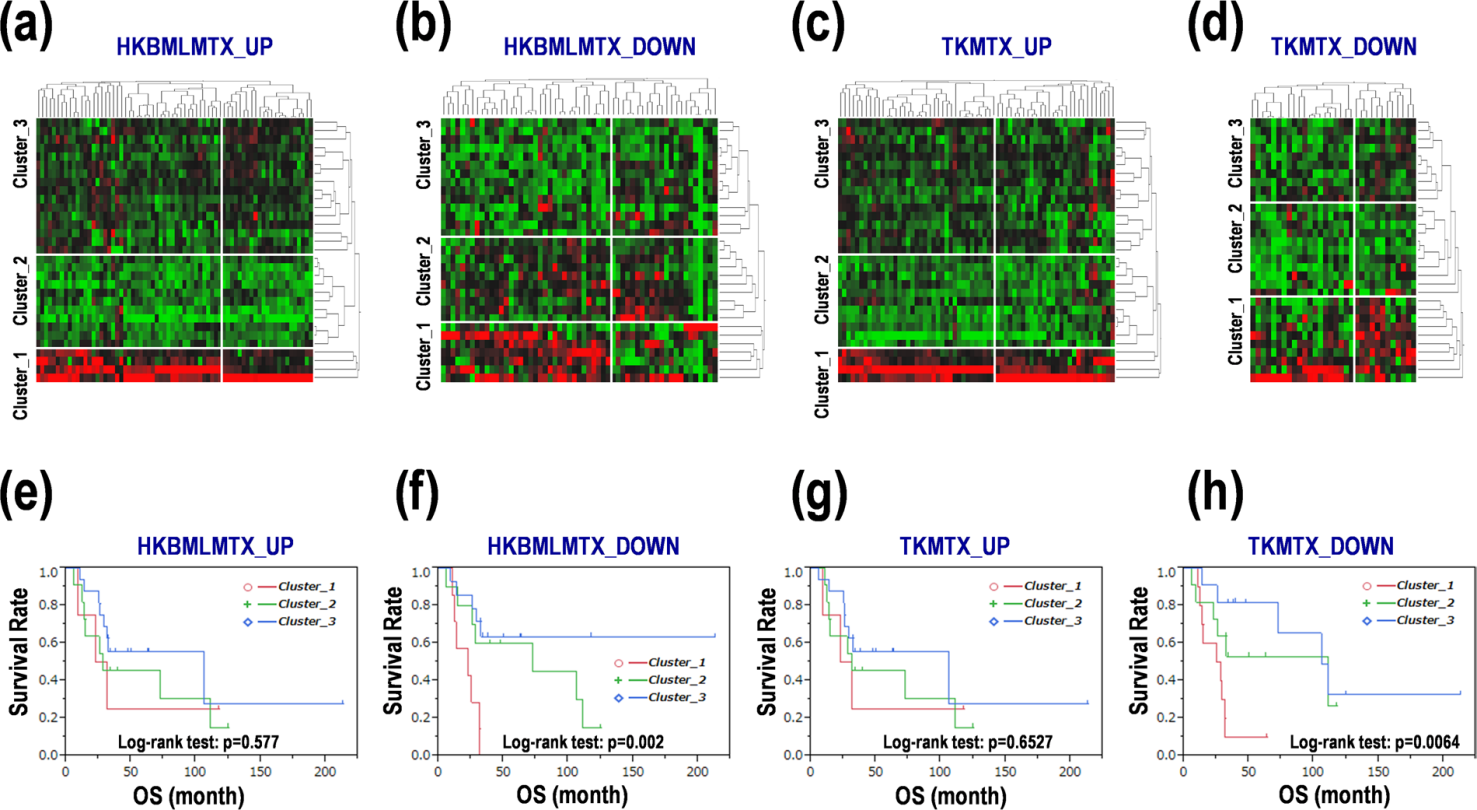
Differential expression of MTX-resistant PCNSL cells-agitated genes in PCNSL with poor prognoses. (**a–d**) Clustering of gene expression of MTX-resistant PCNSL cells in PCNSL specimens. GSEA-assisted upregulated genes in (**a**) HKBML-MTX and (**c**) TK-MTX. GSEA-assisted downregulated genes in (**b**) HKBML-MTX and (**d**) TK-MTX. Green-black-red as low-median-high expression. (**e–h**) Survival distribution of PCNSL patients divided into clusters. Clusters 1, 2, and 3 in each panel of (**e**–**h)**, correspond to those in **(a**–**d)**. Kaplan-Meier survival curves were evaluated with log-rank tests. OS; overall survival.

The above-described results proposed a possibility that GSEA-assisted genes in the MTX-resistant-PCNSL cells were useful for prognosis prediction in PCNSL. We further examined their potentials as prognostic markers in PCNSL. Each clustering was divided PCNSL patients into three clusters, named clusters 1, 2, and 3 in each GSEA-assisted gene category in the MTX-resistant-PCNSL cells (Fig. 3a–d), followed by Kaplan-Meier survival analyses. Survival curves were clearly divided in the clustering of downregulated genes in HKBML-MTX (Table 2, Fig. 3f, Suppl. Fig. S2b) and TK-MTX (Table 2, Fig. 3h, Suppl. Fig. S3b), but not in the clustering of upregulated genes in HKBML-MTX (Table 2, Fig. 3e, Suppl. Fig. S2a) and TK-MTX (Table 2, Fig. 3g, Suppl. Fig. S3a). The GSEA-assisted downregulated genes expressed >3.0-fold (*p* < 0.05) were dnaJ heat shock protein family member B13 (*DNAJB13*), E74-like ETS transcription factor 3 (*ELF3*), glypican 2 (*GPC2*), potassium voltage-gated channel subfamily H member 4 (*KCNH4*), LDL receptor related protein 5 like (*LRP5L*), mitogen-activated protein kinase kinase kinase 15 (*MAP3K15*), RAS guanyl-releasing protein 2 (*RASGRP2*), serpin family A member 10 (*SERPINA10*), solute carrier family 25 member 29 (*SLC25A29*), and src-related kinase lacking C-terminal regulatory tyrosine and N-terminal myristylation sites (*SRMS*) in HKBML-MTX, and kielin cysteine rich bone morphogenetic protein regulator (*KCP*), and purinergic receptor P2X 1 (*P2RX1*) in TK-MTX (Suppl. Table S3). These genes might also be possible prognostic markers in PCNSL.

**Table 2:** Survival analyses for the MTX-resistant cells-promoted genes in PCNSL.

	OS (month)				HR		
	average	sd	median	95% CI	ratio	95% CI	p-value
(HKBML-MTX_UP)							
Cluster_1	23.75	5.6672	27.1	9.1 - NA	1.6823	0.3633–5.9215	0.4666
Cluster_2	53.65	14.1786	28	12.26–111	1.6127	0.586–4.4301	0.3475
Cluster_3	69.4272	11.0503	105.7	25.86 - NA	1		
**(HKBML-MTX_DOWN)**							
Cluster_1	20.9986	3.3749	22.5	10.57–31.23	6.7761	2.0355–24.5467	0.0021
Cluster_2	67.374	14.7578	72.6	5.66–111	1.7979	0.5633–6.1577	0.3196
Cluster_3	28.6114	2.1867	NA	25.86 - NA	1		
**(TK-MTX_UP)**							
Cluster_1	23.75	5.6672	27.1	9.1 - NA	1.6314	0.3524–5.7421	0.4922
Cluster_2	54.6264	13.9255	13.23	12.26–111	1.4997	0.5461–4.1127	0.4241
Cluster_3	68.7559	11.2652	105.7	25.4 - NA	1		
**(TK-MTX_DOWN)**							
Cluster_1	22.742	2.8442	26.565	10.57–31.23	5.7918	1.7729–21.9373	0.0034
Cluster_2	68.0276	15.5806	111	9.1 - NA	1.7116	0.5117–5.9856	0.377
Cluster_3	87.2127	12.4102	105.7	25.4 - NA	1		

Note: OS; overall survival, HR; hazard ratio, sd; standard deviation, CI; confidence interval, NA; not applicable.

### Gene ontology search for the GSEA-assisted genes in MTX-resistant PCNSL cells

Gene ontologies (GOs) of the aforementioned genes were surveyed (*p* < 0.05, Suppl. Table S4). The GOs of commonly upregulated genes in HKBML-MTX and TK-MTX included mitochondrial electron transport (GO 0006123), acetylation (UP KEYWORDS), ubiquitin-like modifier processing (UP KEYWORDS), cardiac muscle contraction (KEGG hsa04260), and some diseases including Parkinson’s (KEGG hsa05012) and Alzheimer’s disease (KEGG hsa05010). The GOs of HKBML-MTX-specifically upregulated genes included acetylation (UP KEYWORDS), splicing (GO 00003898), phosphorylated immunoreceptor tyrosine-based activation motif (*ITAM*) signaling (IPR003110), and a core structure consisting of an open β-barrel with a SH3-like topology (*LSM*) (IPR001163, IPR010920). The GOs of TK-MTX-specifically upregulated genes included oxidative phosphorylation (KEGG bta00190), mitochondrial electron transport (GO 0006123), metal-binding (UP KEYWORDS) and mineral absorption (KEGG bta04978), protein-folding (GO 0006457), and molecular chaperone (UP KEYWORDS). The GOs of HKBML-MTX-specifically downregulated genes included alternative splicing (UP KEYWORDS). The GOs of TK-MTX-specifically downregulated genes included metal-binding (UP KEYWORDS), zinc-finger (UP KEYWORDS), and disulfide bond (UP SEQ FEATURE). These results suggest that a part of upregulated genes in HKBML-MTX and TK-MTX would be involved in mitochondrial electron transport, acetylation, and phosphorylation of proteins, while the downregulated genes would be related to splicing and metal molecular structures in each PCNSL cell.

### Molecular function and signaling pathways in MTX-resistant PCNSL cells

In public data sets including immunologic signatures, oncogenic signatures, computational gene set for cancer modules, and curated gene set for canonical pathways, we further analyzed the GSEA-assisted gene signatures proposed in this study (Suppl. Table S5). The HKBML-MTX-specifically upregulated gene set was correlated to formation of the ternary complex and subsequently 43 S complex (Suppl. Fig. S4a), a cancer module (Suppl. Fig. S4b), thymocytes compared with CD4 T cell in adult blood (Suppl. Fig. S4c), and neutrophil compared with B cell (Suppl. Fig. S4d). The comprehensive results in the GSEA-associated genes in HKBML-MTX suggest that HKBML-type poor prognosis marker candidates in PCNSL would be ribonucleoprotein complex formation, T cell maturation and activation, and cancers including neutrophils. The commonly upregulated gene set in HKBML-MTX and TK-MTX was correlated with a cancer module (Suppl. Fig. S4e), and thymocytes compared with CD4 T cell in adult blood (Suppl. Fig. S4f). Th1 compared with Th17 was detected in TK-MTX-specific cell differentiation (Suppl. Fig. S4g). Considering these results, the GSEA-assisted genes in TK-MTX and HKBML-MTX are similar in gene function. Furthermore, TK-type PCNSL with poor prognoses would also be involved in Th1 maturation from naïve CD4 T cells.

### Estimated molecular networks in the MTX-resistant PCNSL cells and PCNSL tissues

**Figure 4:**
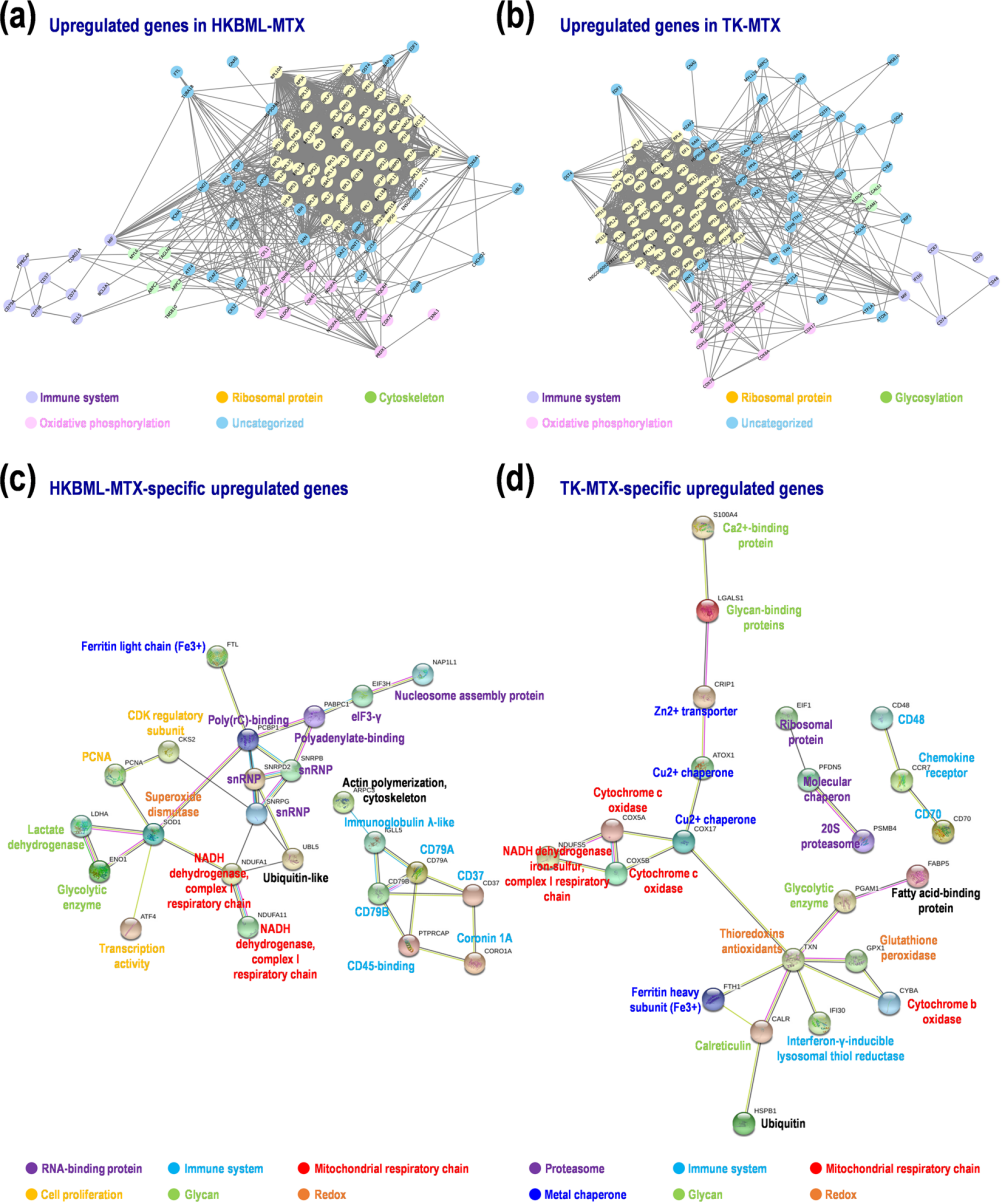
Protein-protein interaction (PPI) networks in MTX-resistant PCNSL cells. Networks were estimated by MCODE and STRING. (**a,b**) PPI networks on upregulated genes in (**a**) HKBML-MTX and (**b**) TK-MTX, compared with control cells. (**c,d**) PPI networks on cell-type-dependent upregulated genes in (**c**) HKBML and (**d**) TK.

Finally, to validate gene function and network in the MTX-resistant PCNSL cells, we investigated protein–protein interactions (PPIs) in the MTX-resistant PCNSL cells and PCNSL tissues (Suppl. Table S6). Here, the 200 upregulated genes in each HKBML-MTX and TK-MTX, and the 300 upregulated genes in PCNSL with poor prognoses were examined. Of these, 58.5–73.6% hit in the database, and 41–59% were found in clusters. Average numbers of edge per node were 37.7–45.7. Of the seed genes as hubs, peroxiredoxin-1 (*PRDX1*) was found in HKBML-MTX and TK-MTX. Nascent-polypeptide-associated complex α polypeptide (*NACA*) was found in TK-MTX and PCNSL with poor prognoses. These results suggest that the MTX-resistant PCNSL cells share a hub of oxidative stress and redox; in addition, TK-MTX and PCNSL with poor prognoses share a hub of ribosome complex and transcription. In HKBML-MTX, PPI hubs for immune system, oxidative phosphorylation, cytoskeleton, and ribosomal protein were detected (Fig. 4a), whereas in TK-MTX, PPI hubs for immune system, oxidative phosphorylation, ribosomal protein, and glycosylation were detected (Fig. 4b). Further, immune system, RNA-binding protein, mitochondria respiratory chain, and cell proliferation were clustered in HKBML-MTX-specific PPI networks (Fig. 4c). While, immune system, proteasome, ATP production with cytochrome c and metal chaperone, redox, and glycoprotein were clustered in TK-MTX-specific PPI networks (Fig. 4d). These results indicate that common PPI networks in both cells are ribosomal protein, immune system, and oxidative phosphorylation, whereas cytoskeleton and glycosylation are unique in HKBML-MTX and TK-MTX, respectively. The distinct PPI networks also included cell proliferation, RNA-binding protein, and NAD + /NADH dehydrogenase in HKBML-MTX, and proteasome, redox, and ATP synthesis with metal chaperone in TK-MTX. In the PCNSL with poor prognoses, PPI hubs with complex networks were involved in immune system, ribosomal protein, mitochondrial protein, and histone, which overlapped PPIs in HKBML-MTX and TK-MTX (Suppl. Fig. S5). These results also suggest that PCNSL includes different cell-types, whereas details for protein expression and interaction should await future studies.

## Discussion

Biallelic inactivation of thymocyte selection-associated high mobility group box (*TOX*) and protein kinase C-δ (*PRKCD*) genes is recurrently found in PCNSL, but not in systemic DLBCL, in addition to a high prevalence of myeloid differentiation primary response 88 (*MYD88*) mutation and cyclin-dependent kinase inhibitor 2 A (*CDKN2A*) biallelic loss^17,18^. In contrast, higher expression of monocyte chemotactic and activating factor-1 (*MCP1*) is observed in PCNSL than DLBCL, which also causes tyrosine phosphorylation of mitogen-activated protein kinase (MAPK) in HKBML *in vitro*^19^. An approach to distinguish PCNSL and non-CNS DLBCL may contribute molecular target therapies. For instance, lenalidomide (TNF-α inhibitor) and rituximab (anti-CD20 monoclonal antibody) are effective in ABC-DLBCL and CNS lymphoma (CNSL), respectively^20^, suggestive of a cell-type-specific drug sensitivity in B-cell lymphoma. In PCNSL, target amplicon exome-sequencing using a cancer-related gene panel detects somatic mutations in the exons of serine-threonine kinase pim-1 (*PIM1*), *MYD88*, *CD79B*, dystonin (*DST*), interferon regulatory factor 4 (*IRF4*), erb-b2 receptor tyrosine kinase 3 (*ERBB3*), myosin heavy chain 11 (*MYH11*), deleted in colorectal carcinoma netrin 1 receptor (*DCC*), and lysine-specific methyltransferase 2D (*KMT2D*)^21^. Hence, data integration and comprehensive interpretation of the current study and previous data would be required for better understanding of PCNSL.

In 64 PCNSL patients, programmed death-ligand 1 (PD-L1) was expressed in 4.1% of PCNSL tumors and 52% of surrounding tissues, and was correlated with interferon (IFN)-γ and CD4 expression in PCNSL cells and microenvironments, respectively^22^. The IFN-γ expression is positively correlated with the CD8 expression^23^. Besides, PD-L1 expression in PCNSL cells elongates OS, whereas PD-L1 expression in microenvironments exhibits an insignificant negative trend with OS^22^. The stimulus-dependent activation of signal transducer and activator of transcription 3 (STAT3) results in increased expression of PD-L1 and PD-L2^23^. Furthermore, soluble factors secreted from TK also induce overexpression of PD-L1, PD-L2, indoleamine 2,3-dioxygenase (IDO1), and cytokines in macrophages surrounding PCNSL cells^23^. Thus, IDO1 expression is positively correlated with expression of macrophage and lymphocyte markers^23^. While, lower immune responses are responsible of poorer prognoses in PCNSL than non-CNS DLBCL^16^. In addition, PCNSL could divide into several subgroups with differential markers including CD208, S100, CD45RO, and human leukocyte antigen (HLA)-DR, which might define *de novo* PCNSL subtypes^16^.

On the other hand, cell-type specificities in refractory recurrence-acquired MTX-resistant PCNSL have not been elucidated. Hard to obtain secondary CNSL specimens and such cell lines for *in vitro* experiments, the MTX-resistant PCNSL cells HKBML-MTX and TK-MTX, and the MTX-resistant non-CNSL cell Raji-MTX, could be useful for *in vitro* models for secondary CNSL and PCNSL with relapse-acquired MTX resistances^13^. B-cell receptor (BCR)/Toll-like receptor (TLR)/nuclear factor κ-light-chain-enhancer of activated B cells (NF-κB) signaling is altered in >90% of PCNSL^17^. Integrated analyses in PCNSL also indicate biases for pathways in immune response, proliferation, apoptosis, and lymphocyte differentiation^17^. Interestingly, in cerebrospinal fluid samples from patients with primary and secondary CNSL, microRNA (miR)-30c acts a diagnostic biomarker^24^. Besides, miR-30d, miR-93, and miR-181b are constituted of prognosis factors in PCNSL tumors^25^. While, miR-101, miR-548b, miR-554, and miR-1202 are associated with cancer immunity in PCNSL tumors^26^. Survivals of PCNSL patients are improved by HD-MTX, and PCNSL located in non-deep structures of brain respond better to HD-MTX alone than that located in deep-structures^27^. In PCNSL, 23 genes for HD-MTX effects have been identified^28^. Among them, *BRCA1* is expressed with the strongest association for OS^28^. Combined MTX-based chemoradiotherapy is a standard first-line treatment for PCNSL, results in a median OS of 25–51 months^29^. However, most PCNSL patients fail the treatment^29,30^. Thus, salvage therapies should be optimized by the chemotherapy regimen for second-line treatments^29,30^. A recent study has demonstrated that IC_50_ for MTX in HKBML-MTX is markedly higher than TK-MTX and Raji-MTX^13^. The gene expression for MTX and folate metabolism is upregulated in both HKBML-MTX and TK-MTX, whereas the expression of *FPGS*, *TYMS*, and *MTHFD1* is upregulated in HKBML-MTX but downregulated in TK-MTX in different manners^13^. In contrast, bortezomib is sensitive in TK-MTX, but not in HKBML-MTX and Raji-MTX, demonstrate PCNSL subtypes^13^.

In this study, using NGS-based global expression profiling of PCNSL patients and MTX-resistant PCNSL cell lines HKBML-MTX and TK-MTX, the GSEA-assisted MTX-resistant genes were examined along OS of PCNSL patients. Thereby, the following potential markers for prognoses in PCNSL were isolated: *FOXD2-AS1*, *MMP19*, *FABP5*, *CD70*, *CLCN2*, *HOXB9*, *INE1*, *LRP5L*, *CSAD*, *MYLK-AS1*, *ZNF169*, *DHDH*, and *IMPA2*. In addition, *NRL*, *PRPF40B*, *RASGRP2*, *RUNX1*, *SMG1P1*, *TOP3B*, *TRIM66*, and *ULK1* were also identified as prognostic marker candidates. Furthermore, the upregulated gene sets in both HKBML-MTX and TK-MTX were involved in ribonucleoprotein complex formation and T cell maturation and activation. HKBML-MTX and TK-MTX gene sets were specifically biased to neutrophils and B cells, and Th17 T cells, respectively, suggest that there are different subtypes in PCNSL. While, PPI networks showed potential hubs for immune system, oxidative phosphorylation, cytoskeleton, and ribosomal protein in both HKBML-MTX and TK-MTX, mitochondria respiratory chain, glycosylation, and cell proliferation in HKBML-MTX, and proteasome, ATP synthesis, and glycoprotein in TK-MTX, suggestive of target therapies corresponding to PCNSL cell-types. Although such genes and signaling pathways should be further examined, these results would help better understanding MTX-refractory relapsed PCNSL.

## Methods

### Tissues

A total of 31 PCNSL patients were enrolled (Suppl. Table S7)^15,31^. Patients were diagnosed according to WHO classification and treated at Toyama Prefectural Central Hospital, Wakayama Medical University School of Medicine, Chiba University, and Yamaguchi University. The study was approved by the Ethics Committee of Kyoto Prefectural University of Medicine (RBMR-G-146), covered recruitments of patients from other centers. Informed consents were obtained from all patients prior to enrollment. Biopsy specimens or resected tumor tissues were immediately snap-frozen. The experiments were performed in accordance with the institutional guidelines.

### Cells

PCNSL cell lines TK and HKBML were purchased from JCRB Cell Bank (National Institutes of Biomedical Innovation, Health and Nutrition) and RIKEN Cell Bank (RIKEN BioResource Center), respectively. TK is ABC-DLBCL but HKBML is unknown (Suppl. Table S8). TK and HKBML were cultured in RPMI 1640 medium with 10% fetal bovine serum (FBS) (Thermo Fisher Scientific) and Ham’s F-12 medium (Nacalai Tesque) with 15% FBS, respectively, in 5% CO_2_ at 37 °C. Methotrexate (MTX)-resistant cells were generated, as described^13–15^. Briefly, TK and HKBML cells were cultured with 1.0 × 10^−6^ M MTX and 1.0 × 10^−7^ M MTX, respectively, for 6 weeks following precultures with lower concentrations of MTX for 9 weeks and 4 weeks, respectively. The MTX-resistant cells were continuously exposed with optimal concentrations of MTX during experiments.

### NGS

NGS was performed, as described^31^. Briefly, total RNA was extracted from biopsy and resected tissues, and PCNSL cells using Isogen II (Nippongene). The quality of RNAs was verified using RNA Pico Chips and Bioanalyzer System (Agilent Technologies). NGS was executed using Illumina HiSeq 2000/2500 platform with a standard 124-bp paired-end read protocol. The arranged sequence data were mapped onto the genome assembly GRCh37/hg19 using TopHat2/Bowtie2^32^. Gene expression levels were estimated as fragments per kilobase of exon per million reads mapped (FPKM). Normalization and detection of DEGs were performed using Cufflinks^33^.

### GSEA

GSEA was performed using GSEAPreranked program, as described^31,34–36^. Briefly, “dataset” calculated from PCNSL patients with good or poor prognosis and “gene sets” derived from PCNSL cells based on NGS were processed with the program. For PCNSL “dataset”, a transcriptome-wide ranking list of gene expression was generated and PCNSL samples were divided into subgroups with good or poor prognosis according to the median OS in the study. Gene symbol and log_2_-fold expression data were sorted in descending order to create the ranking list. For “gene sets” of PCNSL cells, FPKM data was converted into a log_2_-fold ranking list. The top 200 and bottom 200 genes were extracted as the gene sets, respectively. GOs and gene cascades were searched with Database for Annotation, Visualization, and Integrated Discovery (DAVID) and Kyoto Encyclopedia of Genes and Genomes (KEGG), respectively^37–40^.

### Clustering analysis

Expression pattern in each group was clustered into three subgroups with a hierarchical method using the JMP built-in modules (SAS Institute), as described^40–42^.

### Kaplan-Meier survival analysis

Kaplan-Meier analysis was performed with log-rank test to estimate survival distribution in PCNSL subgroups using the JMP built-in modules (SAS Institute), as described^21,40,42^.

### PPI network analysis

PPI was assessed using Search Tool of the Retrieval of Interacting Genes Database (STRING)^43^ and visualized using Cytoscape^44^. Briefly, datasets of the upregulated genes in HKBML-MTX and TK-MTX, compared with HKBML and TK, respectively, were processed to search for hub modules of PPI networks using the Cytoscape plug-in Molecular Complex Detection (MCODE) program, with a degree cutoff = 2, node score cutoff = 0.2, k-core = 2, and max depth = 100. Similarly, datasets of upregulated genes in PCNSLs with poor prognoses, compared with those with good prognoses, were also assessed in PPI networks.

### Statistics

Data was presented as means ± standard deviation of multiple samples and box-whisker plots with the JMP built-in-modules (SAS Institute) and Excel (Microsoft). *p* < 0.05 or false discovery rate (FDR) < 0.01 was considered statistically significant.

## Supplementary information

Supplementary Information.

## Data Availability

The datasets generated during this study are available from the corresponding author on suitable request form.
