# Supplementary material for: GSEA-assisted gene signatures valid for combinations of prognostic markers in PCNSL
Source: Sci Rep. 2020 May 21;10:8435. doi: 10.1038/s41598-020-65463-6 (PMC7242340; doi:10.1038/s41598-020-65463-6)
Supplement: Supplementary file 1 — Supplementary Information. [file 41598_2020_65463_MOESM1_ESM.pdf]

GSEA-assisted gene signatures valid for combinations of prognostic markers in PCNSL

Yasuo Takashima[1,2], Momoko Hamano[3], Junya Fukai[4], Yasuo Iwadate[5], Koji Kajiwara[6], Tsutomu Kobayashi[7], Hiroaki Hondoh[7], and Ryuya Yamanaka[1,2,*]

[1]Osaka Iseikai Clinic for Cancer Therapy, Iseikai Holonics Group, Osaka, Japan

[2]Laboratory of Molecular Target Therapy for Cancer, Graduate School of Medical Science, Kyoto Prefectural University of Medicine, Kyoto, Japan

[3]Department of Bioscience and Bioinformatics, Faculty of Computer Science and Systems Engineering, Kyushu Institute of Technology, Iizuka, Fukuoka, Japan

[4]Department of Neurological Surgery, Wakayama Medical University School of Medicine, Wakayama, Japan

[5]Department of Neurosurgery, Graduate School of Medical Sciences, Chiba University, Chiba, Japan

[6]Department of Neurosurgery, Graduate School of Medical Sciences, Yamaguchi University, Ube, Yamaguchi, Japan

[7]Department of Neurosurgery, Toyama Prefectural Central Hospital, Toyama, Japan

*To whom correspondence should be addressed: ryaman@koto.kpu-m.ac.jp

List of Supplementary Tables

Suppl. Table S1. Summary of GSEA results of the MTX-resistant HKBML and TK cells.

Suppl. Table S2. List of differentially expressed genes with enrichment scores in GSEA.

Suppl. Table S3. Increased expression of MTX-resistance genes in PCNSL with poor prognoses.

Suppl. Table S4. List of gene ontology terms of differentially expressed genes in the MTX-resistant HKBML and TK cells.

Suppl. Table S5. Summary of functional GSEA for the MTX-resistant HKBML and TK cells.

Suppl. Table S6. Summary of the protein-protein interaction analysis.

Suppl. Table S7. Characteristics of the PCNSL patients in the study.

Suppl. Table S8. Characteristics of PCNSL cell lines HKBML and TK.

Supplementary Figure Legends

Suppl. Fig. S1. Precise expression of GSEA-associated marker candidates in PCNSL samples divided by prognoses in box-whisker plots. (**a**) *FOXD2-AS1*, (**b**) *MYLK-AS1*, (**c**) *ZNF169*, (**d**) *DHDH*, and (**e**) *IMPA2* as secondary gene marker candidates for TK-type MTX-resistant PCNSL. FPKM; fragments per kilobase of exon model per million reads mapped.

Suppl. Fig. S2. Gene expression profiling of GSEA-assisted marker candidates in PCNSL samples divided by hierarchical clustering method using the MTX-resistant HKBML-promoted genes. The groups were divided in Cluster 1 and the combined clusters 2 and 3. The results of gene expression patterns in PCNSL were displayed as box-whisker plots. (**a**) GSEA-assisted upregulated genes in the MTX-resistant HKBML. (**b**) GSEA-assisted downregulated genes in the MTX-resistant HKBML. FPKM; fragments per kilobase of exon model per million reads mapped. Representative genes upregulated in Cluster 1 than Clusters 2 and 3 ($p < 0.05$).

Suppl. Fig. S3. Gene expression profiling of GSEA-assisted marker candidates in PCNSL samples divided by hierarchical clustering method using the MTX-resistant TK-promoted genes. The groups were divided in Cluster 1 and the combined clusters 2 and 3. The results of gene expression patterns in PCNSL were displayed as box-whisker plots. (**a**) GSEA-assisted upregulated genes in the MTX-resistant TK. (**b**) GSEA-assisted downregulated genes in the MTX-resistant TK. FPKM; fragments per kilobase of exon model per million reads mapped. Representative genes upregulated in Cluster 1 than Clusters 2 and 3 ($p < 0.05$).

Suppl. Fig. S4. Functional gene set enrichment analysis of the differentially expressed genes in MTX-resistant PCNSL-derived cells in molecular pathways. (**a-d**) GSEA of DEG for HKBML-MTX compared with HKBML in molecular pathways. (**a**) Formation of the ternary complex. (**b**) Cancer module 29. (**c**) Thymocyte vs naïve CD4 T cell. (**d**) Neutrophil vs B cell. (**e-g**) GSEA of DEG for TK-MTX compared with TK in molecular pathways. (**e**) Cancer module 29. (**f**) Thymocyte vs naïve CD4 T cell. (**g**) Th1 vs Th17.

Suppl. Fig. S5. Protein-protein interaction (PPI) networks in PCNSL with poor prognoses. The networks were estimated by the MCODE with the STRING. PPI networks based on the upregulated genes in the PCNSL with poor prognoses, compared with good prognoses.

Suppl. Table S1. Summary of GSEA results of the MTX-resistant HKBML and TK cells.

	SIZE	ES	NES	NOM p-val	FDR q-val	FWER p-val	RANK AT MAX	LEADING EDGE
HKMBLMTX_UP	190	0.4794845	0.9191075	0.6199616	0.7933504	0.631	10075	tags=67%, list=32%, signal=99%
HKMBLMTX_DOWN	149	0.3241223	0.9640492	0.5156576	1	0.585	7631	tags=38%, list=24%, signal=50%
TKMTX_UP	188	0.53476614	1.0430157	0.5122873	1	0.521	8775	tags=63%, list=28%, signal=87%
TKMTX_DOWN	160	0.26003	0.774868	0.79710144	0.7695585	0.781	3330	tags=20%, list=11%, signal=22%

Note: ES; enrichment score, NES; normalized enrichment score. NOM; nominal, FDR; false-discovery rate, FWER; familywise error rate.

Suppl. Table S2. List of diffeentially expressed genes with enrichment scores in GSEA.

GENE SYMBOL	GENE_TITLE	RANK METRIC SCORE	RUNNING ES
(HKMBLMTX_UP)			
NDUFA1	NADH dehydrogenase (ubiquinone) 1 alpha subcomplex, 1, 7.5kDa	0.397140563	-0.026528558
TMSB10	thymosin, beta 10	0.388239563	-0.018115608
SNRPB	small nuclear ribonucleoprotein polypeptides B and B1	0.386943698	-0.007055623
PTPRCAP	protein tyrosine phosphatase, receptor type, C-associated protein	0.340762347	1.51E-04
CKS2	CDC28 protein kinase regulatory subunit 2	0.313993692	0.05327289
MIF	macrophage migration inhibitory factor (glycosylation-inhibiting factor)	0.301482141	0.09182375
HMGN2	high-mobility group nucleosomal binding domain 2	0.293043077	0.09464819
SLC25A5	solute carrier family 25 (mitochondrial carrier; adenine nucleotide translocator), member 5	0.290103614	0.1009331
CD79B	CD79b molecule, immunoglobulin-associated beta	0.284650356	0.113516934
NDUFA11	NADH dehydrogenase (ubiquinone) 1 alpha subcomplex, 11, 14.7kDa	0.282275319	0.13750684
COX8A	cytochrome c oxidase subunit 8A (ubiquitous)	0.2578623	0.17503057
SEC61B	Sec61 beta subunit	0.252367914	0.17725474
UBL5	ubiquitin-like 5	0.240168452	0.20206478
OAZ1	ornithine decarboxylase antizyme 1	0.2371362	0.20612736
EDF1	endothelial differentiation-related factor 1	0.23236765	0.23618126
COX7B	cytochrome c oxidase subunit VIIb	0.229195774	0.24012971
TAGLN2	transgelin 2	0.220478266	0.24467061
ACTG1	actin, gamma 1	0.220252126	0.25109693
COX4I1	cytochrome c oxidase subunit IV isoform 1	0.21883817	0.2557531
GSTP1	glutathione S-transferase pi	0.21809876	0.26173
(HKMBLMTX_DOWN)			
LRP5L	low density lipoprotein receptor-related protein 5-like	0.612622857	0.018523918
FOXD2-AS1		0.529060304	0.029978218
INE1	inactivation escape 1	0.492034793	0.042052694
RASGRP2	RAS guanyl releasing protein 2 (calcium and DAG-regulated)	0.479510814	0.056063123
CSAD	cysteine sulfinic acid decarboxylase	0.447802305	0.06477462
CLCN2	chloride channel 2	0.446014792	0.07958963
BHLHA15		0.418974578	0.08629462
NRL	neural retina leucine zipper	0.413132042	0.098293915
ULK1	unc-51-like kinase 1 (C. elegans)	0.390423298	0.10351291
TOP3B	topoisomerase (DNA) III beta	0.390006036	0.11670284
SLC25A29	solute carrier family 25, member 29	0.379314959	0.124513
ZNF169	zinc finger protein 169	0.374962091	0.13524082
DHDH	dihydrodiol dehydrogenase (dimeric)	0.368844539	0.1446099
ELF3	E74-like factor 3 (ets domain transcription factor, epithelial-specific)	0.36605081	0.1554806
INSL3	insulin-like 3 (Leydig cell)	0.363168061	0.16634868
FAM71F2		0.35780412	0.17559627
MTMR8	myotubularin related protein 8	0.345437855	0.18010959
ZFP92	zinc finger protein 92 homolog (mouse)	0.342328489	0.19010636
TMPRSS13	transmembrane protease, serine 13	0.330932826	0.19482706
HSD3B7	hydroxy-delta-5-steroid dehydrogenase, 3 beta- and steroid delta-isomerase 7	0.329542547	0.2054732
(TKMTX_UP)			
FABP5	fatty acid binding protein 5 (psoriasis-associated)	0.613543928	0.016300282
CD70		0.402614355	-0.005925367
TMSB10	thymosin, beta 10	0.388239563	7.06E-04
EBI3	Epstein-Barr virus induced gene 3	0.378264457	0.007321355
CYBA	cytochrome b-245, alpha polypeptide	0.377113014	0.01841111
IFI30	interferon, gamma-inducible protein 30	0.34098345	0.030232115
LGALS1	lectin, galactoside-binding, soluble, 1 (galectin 1)	0.332313716	0.055619605
FTH1	ferritin, heavy polypeptide 1	0.320709407	0.097788356
MIF	macrophage migration inhibitory factor (glycosylation-inhibiting factor)	0.301482141	0.13193183
COX5B	cytochrome c oxidase subunit Vb	0.300124079	0.13965584
SLC25A5	solute carrier family 25 (mitochondrial carrier; adenine nucleotide translocator), member 5	0.290103614	0.14122255
ATP1A1	ATPase, Na+/K+ transporting, alpha 1 polypeptide	0.259243309	0.19146328
COX8A	cytochrome c oxidase subunit 8A (ubiquitous)	0.2578623	0.2057329
SEC61B	Sec61 beta subunit	0.252367914	0.20794243
CALR	calreticulin	0.238445997	0.2309013
OAZ1	ornithine decarboxylase antizyme 1	0.2371362	0.23667696
PFDN5	prefoldin subunit 5	0.235297084	0.24172507
EDF1	endothelial differentiation-related factor 1	0.23236765	0.27385274
PSMB4	proteasome (prosome, macropain) subunit, beta type, 4	0.231269568	0.28002554
COX7B	cytochrome c oxidase subunit VIIb	0.229195774	0.28485605
(TKMTX_DOWN)			
RUNX1	runt-related transcription factor 1 (acute myeloid leukemia 1; aml1 oncogene)	0.624679029	0.017253693
PRPF40B	PRP40 pre-mRNA processing factor 40 homolog B (S. cerevisiae)	0.525474489	0.026106618
HOXB9	homeobox B9	0.45934996	0.02965762
P2RX1	purinergic receptor P2X, ligand-gated ion channel, 1	0.446724504	0.0406468
MMP19	matrix metallopeptidase 19	0.440925479	0.05302247
TRIM66	tripartite motif-containing 66	0.438422859	0.06580011
IMPA2	inositol(myo)-1(or 4)-monophosphatase 2	0.435186207	0.078190185
PRG2	proteoglycan 2, bone marrow (natural killer cell activator, eosinophil granule major basic protein)	0.428551376	0.08941673
MYLK-AS1		0.393846393	0.090017475
SMG1P1		0.389736831	0.10036442
FAM193B		0.371844381	0.10325697
PSORS1C3	psoriasis susceptibility 1 candidate 3	0.369618744	0.113365814
KCP		0.366103142	0.1226631
CRIP3	cysteine-rich protein 3	0.361417443	0.13156003
FBXO43	F-box protein 43	0.356241137	0.13959408
DOCK6	dedicator of cytokinesis 6	0.348202765	0.14536683
TMEM184A		0.345112145	0.15427125
MASP2	mannan-binding lectin serine peptidase 2	0.344212115	0.16439399
STAG3L1		0.341902554	0.17387024
ZNF514	zinc finger protein 514	0.339674294	0.18305399

Suppl. Table S3. Increased expression of MTX-resistance genes in PCNSL with poor prognoses.

	(HKBMLMTX_UP)		(TKMTX_UP)		(HKBMLMTX_DOWN)		(TKMTX_DOWN)	
GENE	Fold[1]	p-value[2]	Fold	p-value	Fold	p-value	Fold	p-value
ARPC3	3.5641	2.148E-06						
CD37	3.2404	2.596E-06						
CD79A	3.7714	7.647E-05						
CHCHD2	3.2175	1.175E-09	3.2175	1.175E-09				
CKS2	5.9622	3.700E-10						
CORO1A	3.0933	7.271E-07						
COX4I1	4.0134	3.471E-08	4.0134	3.471E-08				
COX6A1	4.1365	1.073E-12	4.1365	1.073E-12				
COX7B	3.8440	9.332E-07	3.8440	9.332E-07				
EIF5A	3.3668	8.085E-11	3.3668	8.085E-11				
ERH	3.8010	1.206E-05	3.8010	1.206E-05				
GAS5	6.2976	2.393E-04	6.2976	2.393E-04				
H2AFZ	3.3518	1.325E-04	3.3518	1.325E-04				
HINT1	4.3028	1.721E-11	4.3028	1.721E-11				
LDHB	5.2468	2.754E-03	5.2468	2.754E-03				
MIF	3.8302	2.649E-10	3.8302	2.649E-10				
MYL6	3.2504	4.291E-09	3.2504	4.291E-09				
NACA	3.6403	1.452E-03	3.6403	1.452E-03				
NDUFA1	3.3906	1.091E-08						
NDUFA11	6.1199	1.049E-06						
OST4	3.0886	3.151E-07	3.0886	3.151E-07				
RAN	3.1684	1.771E-08	3.1684	1.771E-08				
SEC61B	4.8144	3.775E-05	4.8144	3.775E-05				
SLC25A3	3.0109	3.922E-06	3.0109	3.922E-06				
SNHG6	4.6243	8.876E-05						
SNRPB	5.1049	1.082E-10						
SNRPD2	5.3411	7.471E-12						
SNRPG	7.5669	9.708E-08						
TMSB10	4.7962	1.349E-07	4.7962	1.349E-07				
UBA52	4.1412	1.268E-04	4.1412	1.268E-04				
UBL5	6.0870	3.274E-07						
UQCRH	3.7716	9.387E-05	3.7716	9.387E-05				
CD70			4.5856	1.191E-05				
COX5A			3.8030	6.940E-07				
COX5B			3.5677	1.128E-09				
CRIP1			5.1782	9.878E-04				
CYBA			4.8507	7.488E-06				
EBI3			6.2424	2.289E-03				
EIF1			3.5457	1.106E-05				
LGALS1			8.0153	4.765E-06				
PFDN5			5.6825	7.933E-06				
PSMB4			3.1028	3.737E-08				
S100A4			6.5412	1.631E-05				
DNAJB13					4.5702	5.122E-05		
ELF3					4.1088	2.000E-02		
GPC2					3.3392	3.800E-03		
KCNH4					3.0091	3.825E-02		
LRP5L					3.3193	8.830E-06		
MAP3K15					7.1109	9.306E-03		
RASGRP2					3.7308	3.300E-08		
SERPINA10					9.0643	2.732E-02		
SLC25A29					3.2889	4.968E-04		
SRMS					3.4191	3.223E-03		
KCP							4.0113	1.019E-03
P2RX1							6.8758	3.972E-03

Note: [1]Fold (cluster1/cluster2+3)>3.0, [2]$p<0.05$.

Suppl. Table S4. List of gene ontology terms of differentially expressed genes in the MTX-resistant HKBML and TK cells.

Group	Category	Term	Fold Enrichment	PValue	FDR
(COMMON_UP)					
	GOTERM_CC_DIRECT	GO:0070062~extracellular exosome	4.777	7.630E-15	8.737E-12
	UP_KEYWORDS	Acetylation	4.429	5.251E-14	6.029E-11
	GOTERM_CC_DIRECT	GO:0016020~membrane	3.924	3.374E-07	3.850E-04
	GOTERM_CC_DIRECT	GO:0043209~myelin sheath	22.086	5.727E-07	6.533E-04
	GOTERM_CC_DIRECT	GO:0031982~vesicle	22.480	5.752E-06	6.563E-03
	UP_SEQ_FEATURE	cross-link:Glycyl lysine isopeptide (Lys-Gly) (interchain with G-Cter in ubiquitin)	14.493	6.641E-06	7.634E-03
	UP_KEYWORDS	Mitochondrion inner membrane	14.042	7.968E-06	9.146E-03
	GOTERM_BP_DIRECT	GO:1902600~hydrogen ion transmembrane transport	37.200	8.509E-06	1.192E-02
	GOTERM_BP_DIRECT	GO:0006123~mitochondrial electron transport, cytochrome c to oxygen	90.768	1.006E-05	1.409E-02
	GOTERM_MF_DIRECT	GO:0044822~poly(A) RNA binding	4.849	1.447E-05	1.700E-02
	GOTERM_MF_DIRECT	GO:0005515~protein binding	1.662	1.806E-05	2.123E-02
	UP_KEYWORDS	Isopeptide bond	5.263	2.170E-05	2.491E-02
	GOTERM_CC_DIRECT	GO:0031012~extracellular matrix	11.341	2.656E-05	3.030E-02
	GOTERM_MF_DIRECT	GO:0004129~cytochrome-c oxidase activity	60.832	3.476E-05	4.085E-02
	UP_KEYWORDS	Cytoplasm	2.362	5.366E-05	6.158E-02
	KEGG_PATHWAY	hsa05012:Parkinson's disease	11.626	1.082E-04	1.198E-01
	KEGG_PATHWAY	hsa04260:Cardiac muscle contraction	18.344	1.174E-04	1.300E-01
	UP_KEYWORDS	Ubl conjugation	3.812	1.381E-04	1.585E-01
	GOTERM_CC_DIRECT	GO:0005634~nucleus	2.037	1.611E-04	1.836E-01
	KEGG_PATHWAY	hsa05010:Alzheimer's disease	9.827	2.387E-04	2.642E-01
	UP_SEQ_FEATURE	transit peptide:Mitochondrion	7.668	2.302E-04	2.643E-01
	GOTERM_CC_DIRECT	GO:0005743~mitochondrial inner membrane	7.612	2.388E-04	2.721E-01
	GOTERM_BP_DIRECT	GO:0006091~generation of precursor metabolites and energy	34.252	1.969E-04	2.755E-01
	GOTERM_CC_DIRECT	GO:0005751~mitochondrial respiratory chain complex IV	110.672	3.085E-04	3.514E-01
	GOTERM_BP_DIRECT	GO:0098609~cell-cell adhesion	10.048	2.641E-04	3.693E-01
	UP_KEYWORDS	Methylation	4.870	3.334E-04	3.820E-01
	GOTERM_MF_DIRECT	GO:0098641~cadherin binding involved in cell-cell adhesion	9.440	3.522E-04	4.132E-01
	GOTERM_CC_DIRECT	GO:0005829~cytosol	2.459	3.660E-04	4.168E-01
	KEGG_PATHWAY	hsa05016:Huntington's disease	8.599	4.435E-04	4.902E-01
	GOTERM_CC_DIRECT	GO:0005913~cell-cell adherens junction	8.909	4.640E-04	5.281E-01
	UP_KEYWORDS	Mitochondrion	4.356	7.005E-04	8.011E-01
	KEGG_PATHWAY	hsa00190:Oxidative phosphorylation	10.344	1.051E-03	1.158E+00
	GOTERM_CC_DIRECT	GO:0005739~mitochondrion	3.603	1.103E-03	1.251E+00
	KEGG_PATHWAY	hsa04932:Non-alcoholic fatty liver disease (NAFLD)	9.111	1.683E-03	1.849E+00
	UP_KEYWORDS	Phosphoprotein	1.642	1.988E-03	2.259E+00
	UP_KEYWORDS	Transit peptide	6.063	2.575E-03	2.916E+00
	GOTERM_BP_DIRECT	GO:0030036~actin cytoskeleton organization	13.964	2.686E-03	3.698E+00
	GOTERM_BP_DIRECT	GO:0043066~negative regulation of apoptotic process	5.985	2.694E-03	3.708E+00
	GOTERM_CC_DIRECT	GO:0005615~extracellular space	3.204	4.880E-03	5.428E+00
	GOTERM_CC_DIRECT	GO:0005925~focal adhesion	6.133	7.881E-03	8.632E+00
	GOTERM_BP_DIRECT	GO:0002906~negative regulation of mature B cell apoptotic process	226.919	8.549E-03	1.133E+01
	KEGG_PATHWAY	hsa05130:Pathogenic Escherichia coli infection	16.186	1.340E-02	1.389E+01
	UP_KEYWORDS	Actin-binding	7.907	1.299E-02	1.394E+01
	GOTERM_BP_DIRECT	GO:2000343~positive regulation of chemokine (C-X-C motif) ligand 2 production	181.535	1.067E-02	1.396E+01
	KEGG_PATHWAY	hsa05110:Vibrio cholerae infection	15.875	1.391E-02	1.437E+01
	UP_KEYWORDS	Cytoskeleton	3.331	1.505E-02	1.598E+01
	BIOCARTA	h_rhoPathway:Rho cell motility signaling pathway	11.949	2.077E-02	1.697E+01
	GOTERM_CC_DIRECT	GO:0042470~melanosome	14.245	1.786E-02	1.858E+01
	GOTERM_CC_DIRECT	GO:0005737~cytoplasm	1.653	1.880E-02	1.947E+01
	KEGG_PATHWAY	hsa05131:Shigellosis	12.898	2.062E-02	2.061E+01
	GOTERM_BP_DIRECT	GO:0006412~translation	7.175	1.673E-02	2.105E+01
	GOTERM_BP_DIRECT	GO:0032872~regulation of stress-activated MAPK cascade	113.459	1.703E-02	2.138E+01
	GOTERM_MF_DIRECT	GO:0042802~identical protein binding	3.655	2.046E-02	2.157E+01
	KEGG_PATHWAY	hsa00010:Glycolysis / Gluconeogenesis	12.321	2.247E-02	2.226E+01
	GOTERM_BP_DIRECT	GO:0043030~regulation of macrophage activation	100.853	1.913E-02	2.371E+01
	GOTERM_MF_DIRECT	GO:0005200~structural constituent of cytoskeleton	12.443	2.295E-02	2.388E+01
	GOTERM_CC_DIRECT	GO:0005746~mitochondrial respiratory chain	68.511	2.806E-02	2.773E+01
	GOTERM_BP_DIRECT	GO:0019068~virion assembly	75.640	2.543E-02	3.029E+01
	UP_KEYWORDS	Host-virus interaction	5.627	3.157E-02	3.080E+01
	KEGG_PATHWAY	hsa05132:Salmonella infection	9.946	3.344E-02	3.139E+01
	GOTERM_BP_DIRECT	GO:1902166~negative regulation of intrinsic apoptotic signaling pathway in response to DNA damage by p53 class mediator	69.821	2.752E-02	3.236E+01
	GOTERM_BP_DIRECT	GO:0019752~carboxylic acid metabolic process	69.821	2.752E-02	3.236E+01
	GOTERM_BP_DIRECT	GO:0043518~negative regulation of DNA damage response, signal transduction by p53 class mediator	69.821	2.752E-02	3.236E+01
	GOTERM_BP_DIRECT	GO:0050900~leukocyte migration	11.160	2.808E-02	3.290E+01
	KEGG_PATHWAY	hsa04810:Regulation of actin cytoskeleton	5.241	3.541E-02	3.293E+01
	GOTERM_MF_DIRECT	GO:0023026~MHC class II protein complex binding	57.030	3.360E-02	3.308E+01
	GOTERM_BP_DIRECT	GO:0001516~prostaglandin biosynthetic process	64.834	2.961E-02	3.436E+01
	GOTERM_BP_DIRECT	GO:0038096~Fc-gamma receptor signaling pathway involved in phagocytosis	10.721	3.024E-02	3.496E+01
	GOTERM_BP_DIRECT	GO:0050821~protein stabilization	10.011	3.428E-02	3.866E+01
	GOTERM_CC_DIRECT	GO:0005771~multivesicular body	43.598	4.375E-02	3.997E+01
	UP_KEYWORDS	Oxidation	40.119	4.745E-02	4.277E+01
	UP_SEQ_FEATURE	topological domain:Mitochondrial matrix	39.109	4.865E-02	4.364E+01
	GOTERM_MF_DIRECT	GO:0005525~GTP binding	4.753	4.797E-02	4.389E+01
(HKBMLMTX_SPECIFIC_UP)					
	GOTERM_CC_DIRECT	GO:0070062~extracellular exosome	3.456	5.999E-05	6.344E-02
	GOTERM_CC_DIRECT	GO:0005687~U4 snRNP	185.587	1.018E-04	1.077E-01
	SMART	SM00651:Sm	125.723	2.054E-04	1.427E-01
	GOTERM_CC_DIRECT	GO:0005682~U5 snRNP	119.306	2.560E-04	2.705E-01
	INTERPRO	IPR001163:Ribonucleoprotein LSM domain	115.515	2.756E-04	2.880E-01
	GOTERM_CC_DIRECT	GO:0071013~catalytic step 2 spliceosome	29.694	2.804E-04	2.962E-01

Suppl. Table S4. List of gene ontology terms of differentially expressed genes in the MTX-resistant HKBML and TK cells. (Continued)

GOTERM_CC_DIRECT	GO:0005685~U1 snRNP	111.352	2.951E-04	3.117E-01
INTERPRO	IPR010920:Like-Sm (LSM) domain	102.680	3.507E-04	3.664E-01
GOTERM_MF_DIRECT	GO:0044822~poly(A) RNA binding	5.122	4.485E-04	4.292E-01
GOTERM_CC_DIRECT	GO:0005686~U2 snRNP	87.909	4.786E-04	5.051E-01
GOTERM_CC_DIRECT	GO:0019815~B cell receptor complex	371.173	5.164E-03	5.330E+00
GOTERM_CC_DIRECT	GO:0005737~cytoplasm	2.395	6.832E-03	6.995E+00
GOTERM_BP_DIRECT	GO:0000398~mRNA splicing, via spliceosome	22.015	7.408E-03	8.251E+00
KEGG_PATHWAY	cfa03040:Spliceosome	9.100	8.052E-03	8.004E+00
SMART	SM00077:ITAM	209.538	8.781E-03	5.948E+00
INTERPRO	IPR003110:Phosphorylated immunoreceptor signaling ITAM	205.360	9.315E-03	9.331E+00
GOTERM_CC_DIRECT	GO:0034709~methylosome	159.074	1.201E-02	1.200E+01
UP_KEYWORDS	Cytoplasm	7.261	1.524E-02	1.318E+01
UP_KEYWORDS	Phosphoprotein	7.162	1.581E-02	1.364E+01
UP_KEYWORDS	Acetylation	14.246	1.722E-02	1.477E+01
GOTERM_CC_DIRECT	GO:0034719~SMN-Sm protein complex	85.655	2.219E-02	2.113E+01
GOTERM_CC_DIRECT	GO:0071004~U2-type prespliceosome	69.595	2.725E-02	2.534E+01
GOTERM_CC_DIRECT	GO:0046540~U4/U6 x U5 tri-snRNP complex	58.606	3.228E-02	2.932E+01
GOTERM_BP_DIRECT	GO:0000387~spliceosomal snRNP assembly	53.570	3.514E-02	3.392E+01
GOTERM_CC_DIRECT	GO:0005689~U12-type spliceosomal complex	48.414	3.895E-02	3.431E+01
GOTERM_CC_DIRECT	GO:0071011~precatalytic spliceosome	46.397	4.060E-02	3.550E+01
GOTERM_CC_DIRECT	GO:0001772~immunological synapse	38.397	4.886E-02	4.113E+01
(TKMTX_SPECIFIC_UP)				
UP_KEYWORDS	Acetylation	7.445	1.107E-11	1.159E-08
UP_KEYWORDS	Phosphoprotein	3.421	7.499E-05	7.847E-02
GOTERM_BP_DIRECT	GO:0055114~oxidation-reduction process	10.095	2.205E-04	2.636E-01
GOTERM_CC_DIRECT	GO:0070062~extracellular exosome	3.301	2.614E-04	2.443E-01
UP_KEYWORDS	Metal-binding	3.898	4.089E-04	4.272E-01
UP_KEYWORDS	Chaperone	22.831	6.159E-04	6.428E-01
GOTERM_MF_DIRECT	GO:0016531~copper chaperone activity	523.800	3.662E-03	3.516E+00
KEGG_PATHWAY	bta00190:Oxidative phosphorylation	10.786	4.864E-03	4.646E+00
KEGG_PATHWAY	bta04978:Mineral absorption	25.739	5.330E-03	5.080E+00
UP_KEYWORDS	Electron transport	23.087	6.876E-03	6.967E+00
GOTERM_BP_DIRECT	GO:2000510~positive regulation of dendritic cell chemotaxis	212.677	9.011E-03	1.027E+01
GOTERM_BP_DIRECT	GO:0045730~respiratory burst	151.912	1.259E-02	1.407E+01
UP_KEYWORDS	Transport	3.809	1.557E-02	1.515E+01
GOTERM_BP_DIRECT	GO:0006457~protein folding	14.907	1.586E-02	1.741E+01
KEGG_PATHWAY	bta04260:Cardiac muscle contraction	14.156	1.687E-02	1.530E+01
UP_KEYWORDS	Oxidoreductase	6.901	1.758E-02	1.694E+01
GOTERM_BP_DIRECT	GO:0006123~mitochondrial electron transport, cytochrome c to oxygen	88.615	2.150E-02	2.290E+01
GOTERM_CC_DIRECT	GO:0043209~myelin sheath	12.652	2.160E-02	1.848E+01
GOTERM_CC_DIRECT	GO:0005751~mitochondrial respiratory chain complex IV	84.951	2.241E-02	1.911E+01
GOTERM_CC_DIRECT	GO:0045277~respiratory chain complex IV	74.332	2.557E-02	2.152E+01
GOTERM_CC_DIRECT	GO:0009897~external side of plasma membrane	11.363	2.638E-02	2.213E+01
UP_KEYWORDS	Mitochondrion inner membrane	10.646	2.977E-02	2.712E+01
UP_KEYWORDS	Iron	9.738	3.506E-02	3.117E+01
UP_KEYWORDS	Redox-active center	48.923	3.860E-02	3.377E+01
GOTERM_BP_DIRECT	GO:0050766~positive regulation of phagocytosis	48.336	3.907E-02	3.794E+01
GOTERM_CC_DIRECT	GO:0005737~cytoplasm	1.913	4.809E-02	3.694E+01
(HKBMLMTX_SPECIFIC_DOWN)				
UP_KEYWORDS	Alternative splicing	1.404	2.123E-03	2.424E+00
UP_SEQ_FEATURE	splice variant	1.442	1.354E-02	1.568E+01
(TKMTX_SPECIFIC_DOWN)				
UP_KEYWORDS	Metal-binding	2.625	1.144E-03	1.233E+00
UP_KEYWORDS	Zinc	2.817	8.566E-03	8.897E+00
UP_SEQ_FEATURE	disulfide bond	2.372	1.453E-02	1.607E+01
UP_KEYWORDS	Zinc-finger	2.889	2.532E-02	2.426E+01
UP_SEQ_FEATURE	zinc finger region:C2H2-type 7	5.977	2.609E-02	2.712E+01
UP_KEYWORDS	Disulfide bond	2.140	2.646E-02	2.521E+01
UP_SEQ_FEATURE	zinc finger region:C2H2-type 6	5.524	3.193E-02	3.219E+01
UP_KEYWORDS	Signal	1.944	3.245E-02	3.005E+01
UP_SEQ_FEATURE	signal peptide	2.068	3.329E-02	3.332E+01
KEGG_PATHWAY	hsa05310:Asthma	50.956	3.438E-02	2.516E+01
UP_SEQ_FEATURE	splice variant	1.516	3.647E-02	3.590E+01
UP_KEYWORDS	Secreted	2.618	3.869E-02	3.478E+01
UP_SEQ_FEATURE	zinc finger region:C2H2-type 5	5.031	4.040E-02	3.896E+01
UP_SEQ_FEATURE	zinc finger region:C2H2-type 1	4.995	4.114E-02	3.952E+01
UP_SEQ_FEATURE	zinc finger region:C2H2-type 4	4.706	4.769E-02	4.428E+01

Suppl. Table S5. Summary of functional GSEA for the MTX-resistant HKBML and TK cells.

	SIZE	ES	NES	NOM p-val	FDR q-val	FWER p-val	RANK AT MAX	LEADING EDGE
(HKMBLMTX_UP)								
GSE3982_NEUTROPHIL_VS_BCELL_DN	19	0.574	1.611	0.019	0.726	0.261	35	tags=53%, list=18%, signal=58%
GSE1460_DP_THYMOCYTE_VS_NAIVE_CD4_TCELL_ADULT_BLOOD_DN	16	0.558	1.526	0.032	0.692	0.378	67	tags=69%, list=35%, signal=97%
MODULE_29	23	0.632	1.646	0.034	0.041	0.022	41	tags=65%, list=22%, signal=73%
REACTOME_FORMATION_OF_THE_TERNARY_COMPLEX_AND_SUBSEQUENTLY_THE_43S_COMPLEX	29	0.575	1.492	0.067	0.787	0.058	56	tags=66%, list=29%, signal=79%
GSE22886_NAIVE_BCELL_VS_NEUTROPHIL_UP	50	0.472	1.398	0.077	0.864	0.520	63	tags=54%, list=33%, signal=60%
REACTOME_ACTIVATION_OF_THE_MRNA_UPON_BINDING_OF_THE_CAP_BINDING_COMPLEX_AND_EIFS_AND_SUBSEQUENT_BINDING_TO_43S	30	0.561	1.450	0.078	0.525	0.075	56	tags=63%, list=29%, signal=76%
GSE41978_KLRG1_HIGH_VS_LOW_EFFECTOR_CD8_TCELL_DN	54	0.453	1.414	0.081	1.000	0.503	78	tags=61%, list=41%, signal=74%
(TKMTX_UP)								
GSE26030_TH1_VS_TH17_DAY15_POST_POLARIZATION_DN	15	0.543	1.562	0.012	0.620	0.334	71	tags=67%, list=37%, signal=98%
GSE1460_DP_THYMOCYTE_VS_NAIVE_CD4_TCELL_ADULT_BLOOD_DN	21	0.529	1.597	0.040	0.896	0.280	65	tags=62%, list=34%, signal=83%
MODULE_29	24	0.599	1.579	0.051	0.270	0.076	50	tags=67%, list=26%, signal=79%
GSE3982_NEUTROPHIL_VS_BCELL_DN	19	0.528	1.466	0.067	0.813	0.440	44	tags=53%, list=23%, signal=62%
(HKMBLMTX_DOWN)								
none of the gene sets passed size thresholds								
(TKMTX_DOWN)								
none of the gene sets passed size thresholds								

Note: ES; enrichment score, NES; normalized enrichment score. NOM; nominal, FDR; false-discovery rate, FWER; familywise error rate.

Suppl. Table S6. Summary of the protein-protein interaction analysis.

	HKBML-MTX (%)	TK-MTX (%)	PCNSL POOR (%)
Genes entered	200 (100)	200 (100)	300 (100)
Genes detected	122 (61)	117 (58.5)	221 (73.6)
Genes clustered	92 (46)	82 (41)	177 (59)
Numbers of cluster	11	5	14
Seed genes	4 (2)	5 (2.5)	12 (4)
Clustered genes	79 (39.5)	77 (38.5)	165 (55)
Unclustered genes	9 (4.5)	0 (0)	0 (0)
Numbers of edge	5236	4422	10101
Median	62.5	55	32
Ave	42.9	37.7	45.7
max	77	69	115
Min	1	1	1

Suppl. Table S7. Characteristics of the PCNSL patients in the study.

				Univariable analysis		Multivariable analysis	
		N (%)	OS[a] (days): Median (Min–Max)	HR[b] (95% CI[c])	*p*-value	HR (95% CI)	*p*-value
Total		31 (100)	765 (188 - 3611)				
Age	Age < 60	12 (38.7)	994.5 (317.1 – 6378)	1.0		1.0	
	Age > 60	19 (61.2)	936.9 (169.8 – 3738)	1.23 (0.49 – 3.31)	0.65	0.79 (0.18 – 3.20)	0.73
Gender	Male	16 (51.6)	990 (169.8 – 6378)	1.0		1.0	
	Female	15 (48.3)	951 (273 – 3738)	0.45 (0.11 – 1.52)	0.2	0.59 (0.19 – 1.74)	0.59
KPS[d]	KPS 0 – 60	19 (61.2)	936.9 (169.8 – 317.1)	1.0		1.0	
	KPS 70 – 100	12 (38.7)	1080 (317.1 – 6378)	0.42 (0.13 – 1.16)	0.09	0.24 (0.008 – 6.56)	0.34
MSKCC[e]	MSKCC 1	4 (14.8)	1515 (840 – 3330)	1.0		1.0	
	MSKCC 2	9 (29.0)	951 (317.1 – 6378)	1.19 (0.25 – 8.36)	0.83	2.05 (0.20 – 48.22)	0.55
	MSKCC 3	18 (58.0)	948.45 (169.8 – 3171)	2.22 (0.59 – 14.56)	0.26	0.93 (0.10 – 20.66)	0.95
IELSG[f]	IELSG 0 - 1	6 (19.3)	2175 (317.1 – 6378)	1.0		1.0	
	IELSG 2 - 3	20 (64.5)	807.9 (169.8 – 3525)	2.75 (0.81 – 12.82)	0.1	3.42 (0.63 – 24.15)	0.15
	IELSG 4 - 5	5 (16.1)	1429.2 (936.9 – 3171)	1.51 (0.26 – 8.61)	0.62	0.86 (0.07 – 11.91)	0.91
Chemotherapy	Ionizing radiation	3 (9.6)	762 (169.8 – 775.8)	1.0		1.0	
	Polychemotherapy	9 (29.0)	1429.2 (317.1 – 6378)	0.24 (0.05 – 1.25)	0.08	0.49 (0.04 – 4.33)	0.52
	HD-MTX[g]	19 (61.2)	969 (273 – 3525)	0.20 (0.05 – 0.98)	0.04	0.24 (0.04 – 1.43)	0.11

Note: [a]OS; overall survival, [b]HR; hazard ratio, [c]CI; confidence interval, [d]KPS; Karnofsky Performance score, [e]MSKCC; Memorial Sloan Kettering Cancer Center risk score, [f]IELSG; International Extranodal Lymphoma Study Group risk score, [g]HD-MTX; high-dose methotrexate; $p < 0.05$, statistically significant.

Suppl. Table S8. Characteristics of PCNSL cell lines HKBML and TK.

	Age (years)	Gender	Primary/secondary	Histology	HIV	EBV
HKBML	NA	Male	primary	NA	(-)	(+)
TK	22	Male	primary	ABC-DLBCL	(-)	(-)

Note: (+); positive, (-); negative, HIV; human immunodeficiency virus, EBV; Epstein-Barr virus, ABC; activated B-cell-like subtype, NA; not applicable.

Suppl. Fig. S1

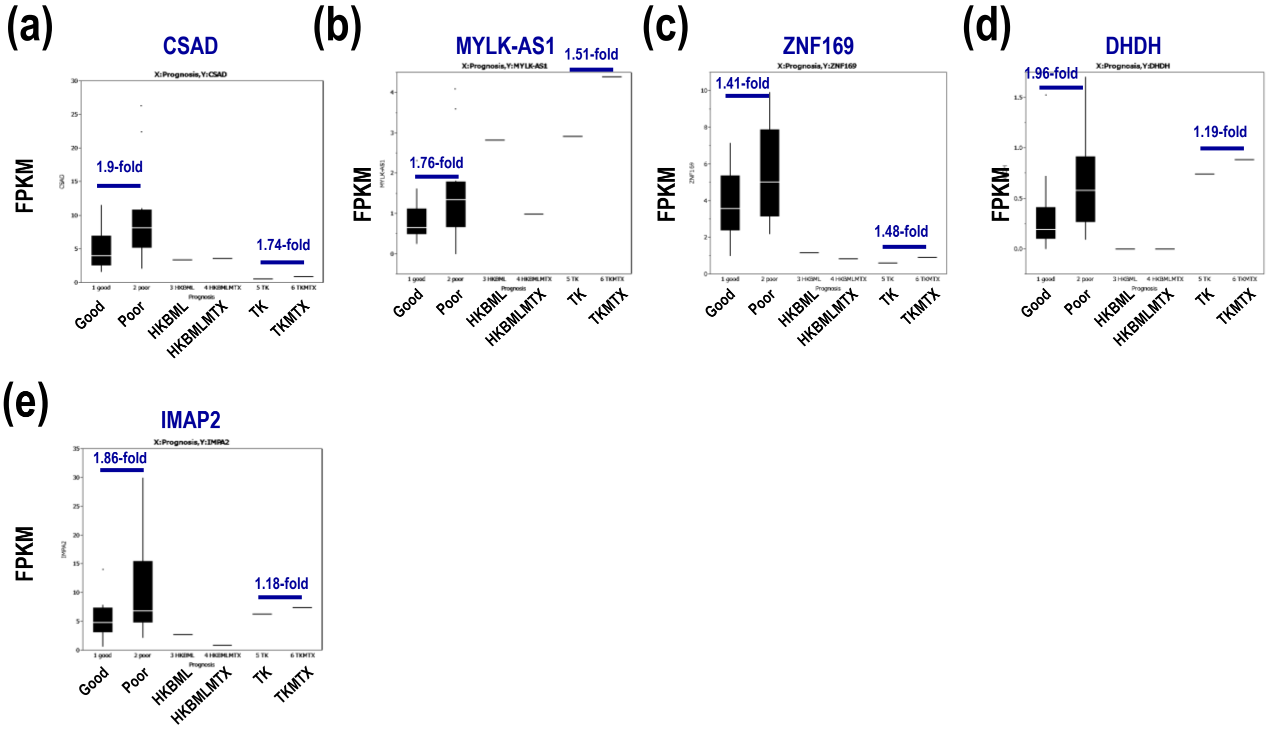

Suppl. Fig. S1. Precise expression of GSEA-associated marker candidates in PCNSL samples divided by prognoses in box-whisker plots. (**a**) FOXD2-AS1, (**b**) MYLK-AS1, (**c**) ZNF169, (**d**) DHDH, and (**e**) IMAP2 as secondary gene marker candidates for TK-type MTX-resistant PCNSL. FPKM; fragments per kilobase of exon model per million reads mapped.

Suppl. Fig. S2

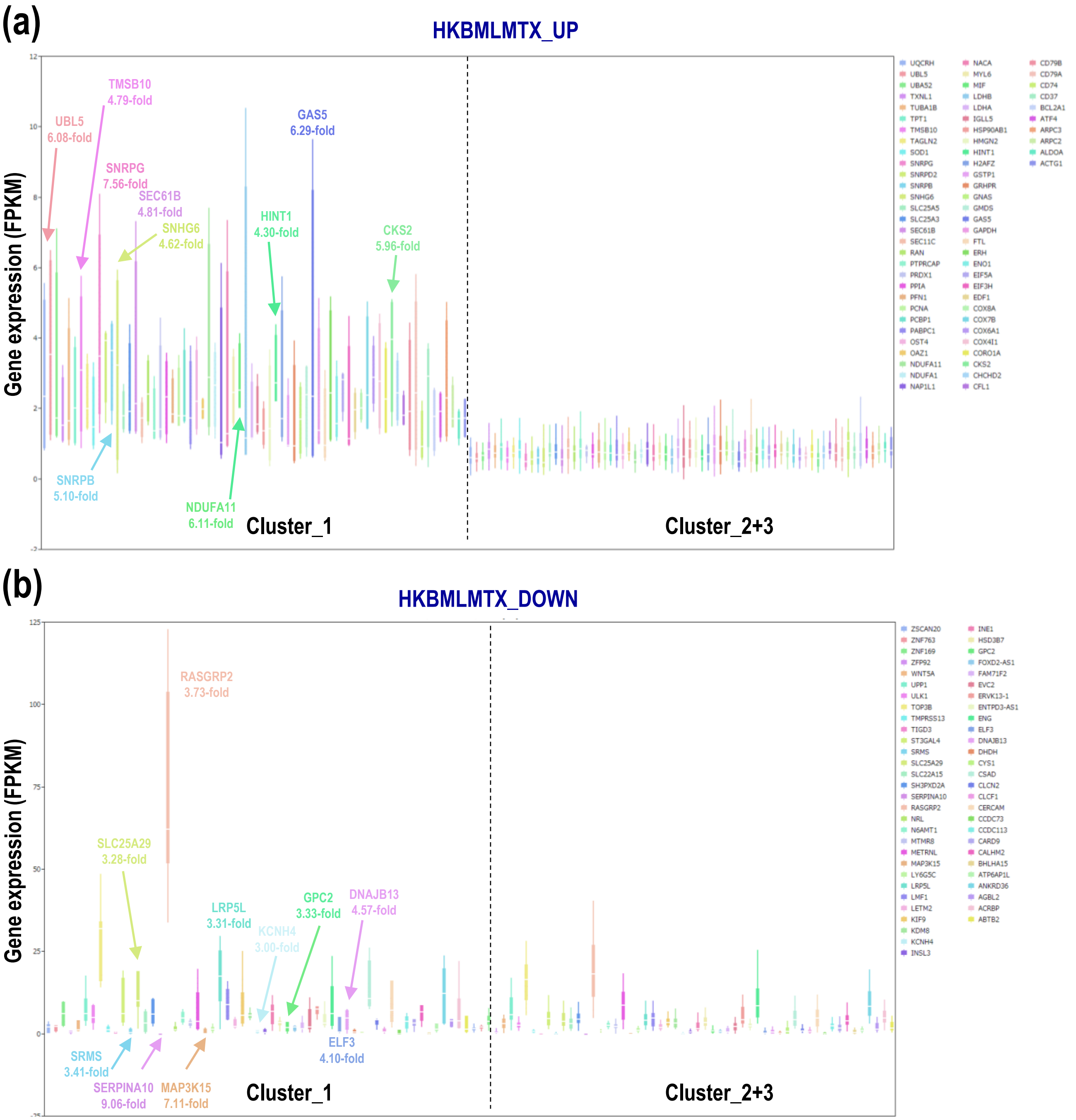

Suppl. Fig. S2. Gene expression profiling of GSEA-assisted marker candidates in PCNSL samples divided by hierarchical clustering method using the MTX-resistant HKBML-agitated genes. The groups were divided in Cluster 1 and the combined clusters 2 and 3. The results of gene expression patterns in PCNSL were displayed as box-whisker plots. (**a**) GSEA-assisted upregulated genes in the MTX-resistant HKBML. (**b**) GSEA-assisted downregulated genes in the MTX-resistant HKBML. FPKM; fragments per kilobase of exon model per million reads mapped. Representative genes upregulated in Cluster 1 than Clusters 2 and 3 (p < 0.05).

Suppl. Fig. S3

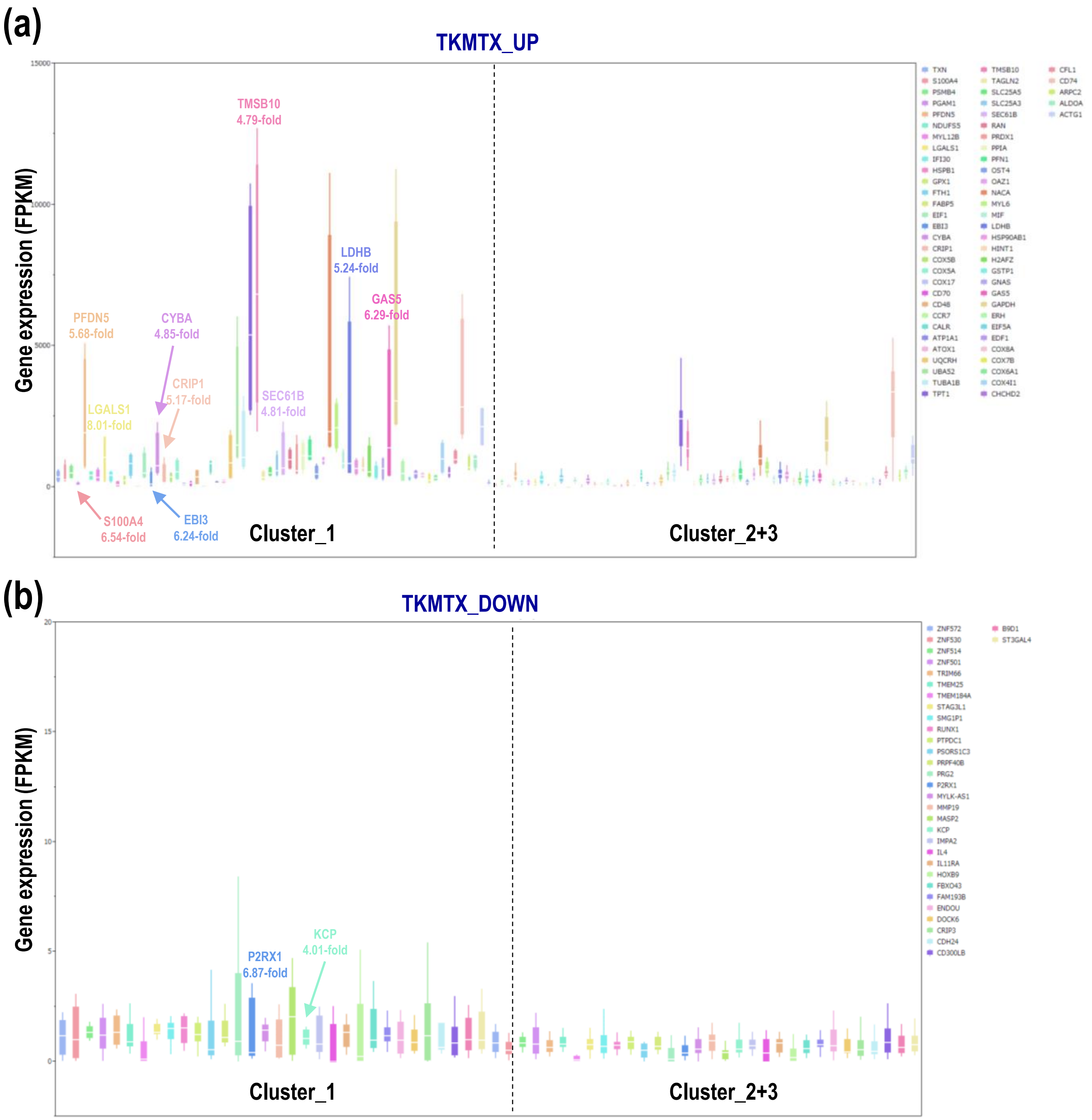

Suppl. Fig. S3. Gene expression profiling of GSEA-assisted marker candidates in PCNSL samples divided by hierarchical clustering method using the MTX-resistant TK-agitated genes. The groups were divided in Cluster 1 and the combined clusters 2 and 3. The results of gene expression patterns in PCNSL were displayed as box-whisker plots. (**a**) GSEA-assisted upregulated genes in the MTX-resistant TK. (**b**) GSEA-assisted downregulated genes in the MTX-resistant TK. FPKM; fragments per kilobase of exon model per million reads mapped. Representative genes upregulated in Cluster 1 than Clusters 2 and 3 ($p < 0.05$).

Suppl. Fig. S4

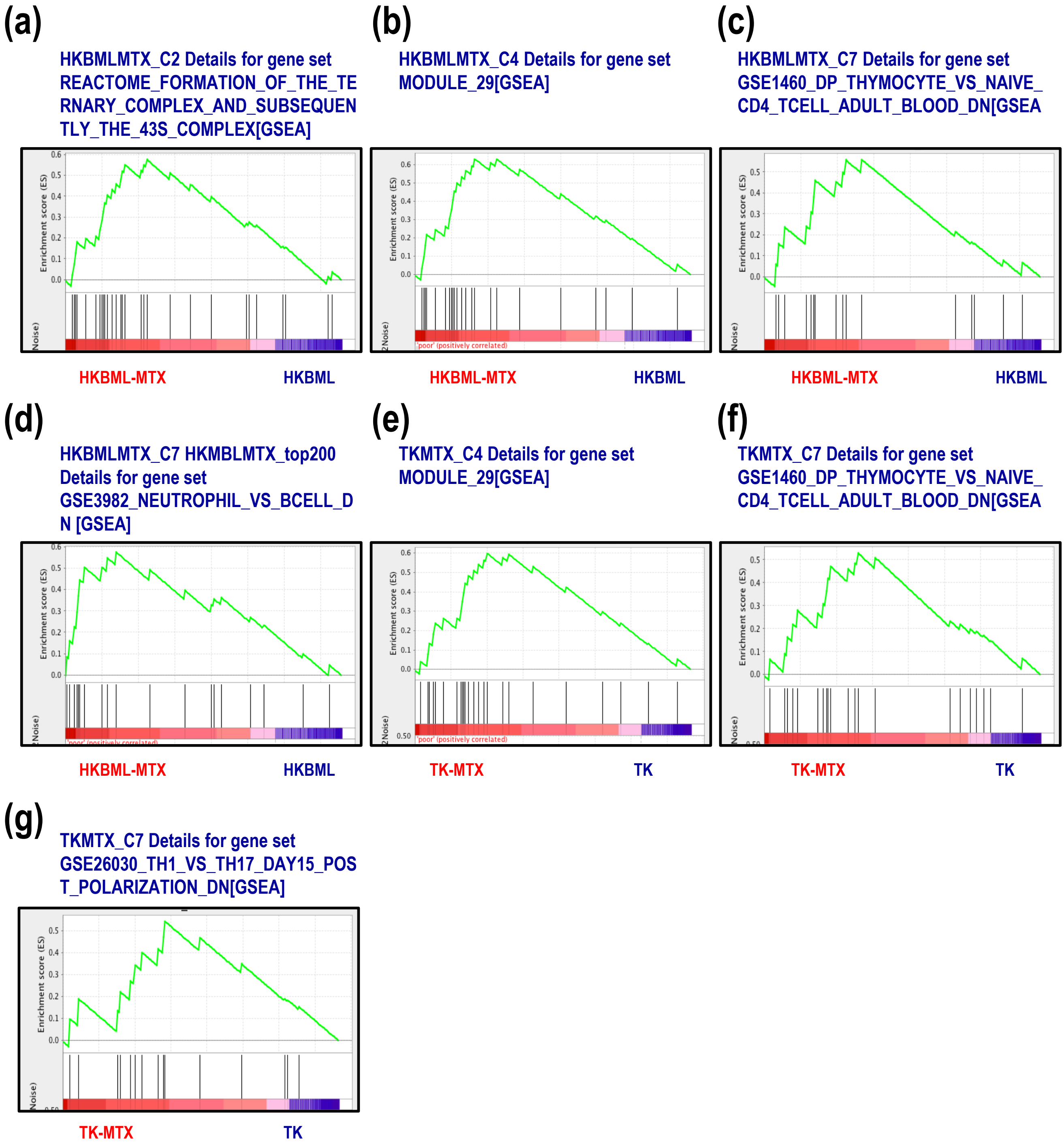

Suppl. Fig .S4. Functional gene set enrichment analysis of the differentially expressed genes in MTX-resistant PCNSL-derived cells in molecular pathways. (**a-d**) GSEA of DEG for HKBML-MTX compared with HKBML in molecular pathways. (**a**) Formation of the ternary complex. (**b**) Cancer module 29. (**c**) Thymocyte vs naïve CD4 T cell. (**d**) Neutrophil vs B cell. (**e-g**) GSEA of DEG for TK-MTX compared with TK in molecular pathways. (**e**) Cancer module 29. (**f**) Thymocyte vs naïve CD4 T cell. (**g**) Th1 vs Th17.

Suppl. Fig. S5

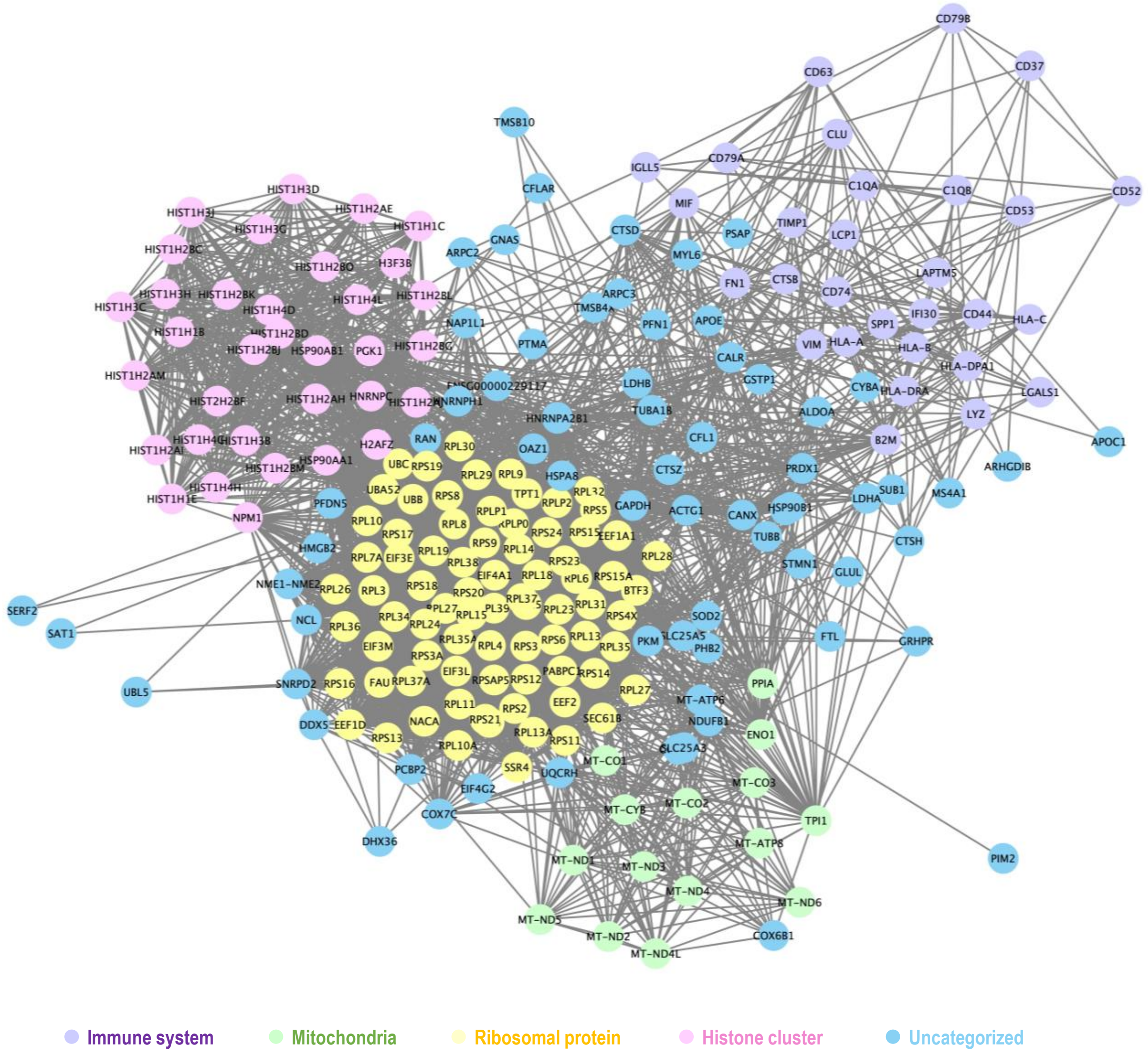

Suppl. Fig. S5. Protein-protein interaction (PPI) networks in PCNSL with poor prognoses. The networks were estimated by the MCODE with the STRING. PPI networks based on the upregulated genes in the PCNSL with poor prognoses, compared with the good prognoses.
